# Systemic *Listeria monocytogenes* infection in aged mice induces long-term neuroinflammation: the role of miR-155

**DOI:** 10.1186/s12979-022-00281-0

**Published:** 2022-05-25

**Authors:** Benjamin R. Cassidy, William E. Sonntag, Pieter J. M. Leenen, Douglas A. Drevets

**Affiliations:** 1 Infectious Diseases, Department of Internal Medicine, 800 Stanton L. Young, Suite 7300, Oklahoma City, OK 73104 USA; 2 Department of Biochemistry and Molecular Biology, University of Oklahoma Health Sciences Center, Oklahoma City, OK USA; 3 Department of Immunology, Erasmus University Medical Center, Rotterdam, The Netherlands

**Keywords:** *Listeria*, miR-155, Bacterial meningitis, sepsis, Tissue-resident memory T cells, Neuroinflammation

## Abstract

**Background:**

Understanding mechanisms of pathologic neuroinflammation is essential for improving outcomes after central nervous system infections. Brain tissue-resident memory T cells (bT_RM_) are recruited during central nervous system infection and promote pathogen control as well as noxious inflammation. Our prior studies in young mice showed optimal recruitment of CD8^+^ bT_RM_ during neuroinvasive *Listeria monocytogenes* (*Lm*) infection required miR-155, and was significantly inhibited by anti-miR-155 oligonucleotides. Since *Lm* is an important pathogen in the elderly, we hypothesized anti-miR-155 would also inhibit accumulation of CD8^+^ bT_RM_ in aged mice infected with *Lm*.

**Methods:**

Young (2 mo) and aged (> 18 mo) male C57BL/6 mice were infected intra-peritoneally with wild-type *Lm*, or avirulent *Lm* mutants lacking the genes required for intracellular motility (Δ*actA*) or phagosomal escape (Δ*hly*), then were given antibiotics. Brain leukocytes and their intracellular cytokine production were quantified by flow cytometry >28d post-infection (p.i.). The role of miR-155 was tested by injecting mice with anti-miR-155 or control oligonucleotides along with antibiotics.

**Results:**

Aged mice had significantly more homeostatic CD8^+^ bT_RM_ than did young mice, which did not increase after infection with wild-type *Lm* despite 50% mortality, whereas young mice suffered no mortality after a larger inoculum. For direct comparison of post-infectious neuroinflammation after the same inoculum, young and aged mice were infected with 10^7^ CFU Δ*actA Lm*. This mutant caused no mortality and significantly increased CD8^+^ bT_RM_ 28d p.i. in both groups, whereas bone marrow-derived myeloid cells, particularly neutrophils, increased only in aged mice. Notably, anti-miR-155 reduced accumulation of brain myeloid cells in aged mice after infection, whereas CD8^+^ bT_RM_ were unaffected.

**Conclusions:**

Systemic infection with *Lm* Δ*actA* is a novel model for studying infection-induced brain inflammation in aged mice without excessive mortality. CD8^+^ bT_RM_ increase in both young and aged mice after infection, whereas only in aged mice bone marrow-derived myeloid cells increase long-term. In aged mice, anti-miR-155 inhibits brain accumulation of myeloid cells, but not CD8^+^ bT_RM_. These results suggest young and aged mice differ in manifestations and mechanisms of infection-induced neuroinflammation and give insight for developing therapies to ameliorate brain inflammation following severe infection in the elderly.

**Supplementary Information:**

The online version contains supplementary material available at 10.1186/s12979-022-00281-0.

## Background

Activation of inflammatory pathways in the brain is a root cause of infection-induced neurological injury [1, 2]. Understanding how to interrupt pathologic brain inflammation is a pressing global healthcare need as meningitis and encephalitis rank as the 4th and 10th largest contributors, respectively, among all neurological disorders to age-standardized disability-adjusted life-years [3]. During acute central nervous system (CNS) infection, bone marrow-derived leukocytes enter the CNS and produce pro-inflammatory mediators, e.g. IL-1β, IFN-γ and TNF, as well as chemokines that amplify the inflammatory response [4–6]. Most populations of recruited leukocytes return to pre-infection levels after the infection is cleared [7]. However, elevated numbers of CD8^+^ tissue-resident memory cells (T_RM_) remain in the brain after CNS infection with bacteria, protozoans, and viruses [8–12]. These long-lived, non-recirculating T-lymphocytes also accumulate in the brain during to non-infectious conditions such Alzheimer’s disease and multiple sclerosis, as well as during normative aging [13–17]. Infection-induced brain CD8^+^ bT_RM_ (bT_RM_) assist with rapid pathogen removal and immune surveillance, but also drive pathologic inflammation, gliosis, and neuro-cognitive dysfunction via secreting IFN-γ [9, 18]. In contrast, the biological functions of aging-induced CD8^+^ bT_RM_ remain unknown. These cells are expanded clonally and phenotypically distinct from CD8^+^ T-lymphocytes in the blood, and produce pro-inflammatory cytokines upon activation and inhibit neural stem cell proliferation via secretion of IFN-γ [15–17].

Corticosteroids are the principal drugs used to reduce brain inflammation in patients with bacterial CNS infections [19]. Although helpful, these drugs reduce mortality only in select groups of patients infected with certain organisms, and do not improve long-term neurological outcomes in adults with bacterial meningitis [20–22]. Experimental therapies such as thalidomide and glycerol also do not improve outcomes in tubercular or bacterial meningitis, respectively [23, 24]. Thus, new treatments are needed to reduce post-infectious CNS inflammation and its downstream complications. This is particularly true in the elderly, who have worse outcomes after CNS infection compared to younger individuals [22, 25–27].

Infection of mice with *L. monocytogenes (Lm)* is an established model for the study of CNS inflammation [28]. *Lm* is a neuroinvasive, facultative intracellular bacterium that causes sepsis and CNS infection in humans, with high mortality rates and long-term neurological sequelae in 44% of survivors [22, 29]. *Lm* is a highly relevant pathogen of the aged as the incidence of invasive listeriosis and risk of death from it increase with years of age independent of co-existing conditions or immune suppression [22, 26, 30]. To study long-term inflammatory changes in the brain, we previously adapted the mouse model by treating *Lm*-infected animals, which would otherwise die of infection, with the same antibiotic used in humans [6, 19]. We identified significantly increased numbers of CD8^+^ bT_RM_ 28 days after neuroinvasive *Lm* infection in young mice [8]. These cells were extravascular and had a CD3^+^CD8^+^CD44^+^CD62L^−^CD69^+^CX3CR1^−^ phenotype characteristic of T_RM_. Importantly, accumulation of CD8^+^ bT_RM_ was significantly lower in miR-155^−/−^ mice, and also significantly suppressed by administration of anti-miR-155 oligonucleotide in wild-type mice suggesting this could be a novel anti-inflammatory therapy.

In the current study, we aimed to elucidate the consequences of age-related changes in *Lm* CNS infection in greater detail. In particular, experiments presented here sought to determine if *Lm* infection induced differential accumulation of CD8^+^ bT_RM_ in young and aged mice, and if so, to test the degree to which anti-miR-155 inhibited their accumulation. The challenge of aged mice being more susceptible to *Lm* infection than are young mice was overcome by using avirulent *Lm* Δ*actA* mutants that are deficient in intracellular motility, but escape phagosomes and trigger cytosolic surveillance mechanisms and neuroinflammation [6, 31, 32]. Results using this infection model clearly demonstrate that numbers of CD8^+^ bT_RM_ increase in aged mice after systemic infection. Importantly, we also identified fundamental differences between young and aged mice in the types of bone marrow-derived cells that contribute to post-infectious neuroinflammation, and the molecular mechanisms controlling them. Elucidating these pathways is essential for developing new therapies to improve survival, and lessen the neurological and neuropsychiatric complications in survivors of sepsis and CNS infections, which are particularly harsh in the elderly.

## Methods

### Antibodies

Fluorochrome-conjugated mAb (clone, fluorochrome) and isotype-matched control antibodies were purchased from BD Pharmingen (San Diego, CA): CD62L (MEL-14, BV510), CD44 (IM7, PE-CF594), or from Biolegend (San Diego, CA): CD11b (M1/70, BV421), CD3 (17A2, PE), CD8a (53–6.7, Alexa Fluor 488), CD4 (RM4–5/GK1.5, BV605 and BV785), CD45 (30-F11, PE/Cy7), CX3CR1 (SA011F11, BV605), Ly-6G (1A8, BV510), Ly-6C (HK1.4, PerCP/Cy5.5), CD69 (H1.2F3, BV711), CD103 (2E7, APC), IFN-γ (XMG1.2, BV421), Granzyme B (QA16A02, APC), and TNF (MP6-XT22, BV605).

### Bacteria

*Lm* strain EGD was originally obtained from P. Campbell [33]. Strain 10,403 s was obtained from the American Type Culture Collection (Manassas, VA). *Lm* mutants constructed from the 10,403 s parent strain deficient in actA (Δ*actA*) DP-L1942 and listeriolysin O (Δ*hly*) DP-L2161 were generous gifts from D. Portnoy (Univ. of California, Berkeley, CA) [34, 35]. Bacterial cultures were stored in brain-heart infusion (BHI) broth (Difco, Detroit, MI) at 10^9^ CFU/ml at − 80 °C. Prior to experiments, 10 μl of the stock culture was diluted 1:10,000 in BHI and was cultured overnight at 37 °C with shaking.

### Animal infection and antibiotic treatment

C57BL/6 J mice were purchased from Jackson Laboratories (Bar Harbor, ME). C57BL/6 N mice were purchased from Charles River Laboratories (Wilmington, MA). C57BL/6JN mice were obtained from the National Institute on Aging (NIA) Aged Rodent Colonies. Age and genotype of mice used in experiments are given in the figure captions. Male mice were used in all experiments with the exception that four females were used in experiments reported in Fig. 5. Mice were infected i.p. with the indicated amount and strain of *Lm* in a total volume of in 500 μL PBS. CFU bacteria in all inocula were quantified by serial dilution and plating on agar after injection. Mice were injected i.p. with 2 mg ampicillin (Butler Schein Animal Health, Dublin, OH) three times at 10–12-h intervals beginning 48 h p.i., then received amoxicillin (2 mg/mL final concentration) added to their drinking water beginning 3d p.i., which continued until d16 p.i. when mice had received a total of 14 days treatment with antibiotics [6, 8]. Because i.p. ampicillin was deemed unnecessary for survival in aged mice infected with *Lm* Δ*actA* and that fewer injections would minimize distress, animals used for experiments reported in Figs. 6, 7 and 8 only received amoxicillin in their drinking water 3-16d p.i. All mice were ear-tagged and weighed daily for 14d. Uninfected mice received the same course of antibiotics as infected mice in any given experiment. Mice were euthanized by CO_2_ asphyxiation and exsanguinated via femoral vein cut-down, then were perfused trans-cardially with 25 mL iced, sterile PBS containing 2 U/ml heparin. After perfusion, brain, spleen and liver were removed aseptically at necropsy. Organs, or organ portions used for culture, were weighed then homogenized in sterile ddH_2_O. CFU *Lm* were quantified by serial 10-fold dilutions on tryptic soy agar followed by incubation at 37 °C for 24 h. Mouse experiments were approved by the Institutional Animal Care and Use Committee (IACUC) of the University of Oklahoma Health Sciences Center (OUHSC).

### Oligonucleotides

miRCURY Locked Nucleic Acid™ (LNA) miRNA inhibitor oligonucleotides were custom ordered from Qiagen (Hilden, Germany). Anti-miR-155 (Product MMU-MIR-155-5P INH, Cat. No. 339203 YCI0200322-FZA, sequence 5′-3′ TCACAATTAGCATTA) and Negative Control A “LNA scramble” (Product NEGATIVE CONTROL A, Cat. No. 339203 YCI0200319-FZA, sequence 5′-3′ ACGTCTATACGCCCA) were used according to the manufacturer’s guidelines. After receipt, oligonucleotides were re-suspended in 1x PBS to a final concentration of 2 mg/mL and frozen in aliquots at − 80 °C until use. They were injected subcutaneously (s.c.) into infected mice at a dose of 20 mg/kg body weight 2d, 4d, 6d, and 8d p.i. as previously described [8].

### Brain digestion

Perfused brains were digested enzymatically by incubation in Miltenyi C tubes (Miltenyi Biotec, San Diego, CA) containing 0.5 mg/mL Collagenase IV and 0.025 mg/mL DNAse I in RPMI-1640 (ATCC, Manassas, VA) plus 1% penicillin/streptomycin and 10% fetal bovine serum (FBS) for 45 min at 37 °C. Dissociated tissue was filtered through a 70 nm cell strainer with 10 mL HBSS without Ca^++^/Mg^++^ (Lonza, Basel, Switzerland) then centrifuged at 300x g for 10 min at room temperature (RT). The supernatant was discarded, and myelin was removed by resuspending the cells in 30% Percoll (GE Healthcare, Chicago, IL) in a 15 mL polypropylene conical tube followed by centrifugation at 700x g at RT for 10 min. The cell pellet was washed with PBS + 0.5% BSA, and then incubated in RBC Lysis Buffer (Life Technologies Corp., Carlsbad, CA) for 5 min at RT. Leukocytes were washed twice with PBS + 0.5% BSA at 300x g for 10 min at 4 °C then re-suspended in 3 mL of PBS + 0.5% BSA and counted using the Countess II FL Automated Cell Counter (Life Technologies Corp.).

### Flow cytometry

Cells were incubated on ice with 2 μL of anti-CD16/32 TruStain fcX (BioLegend, San Diego, CA) plus 10 μL of Brilliant Stain Buffer Plus (BD Biosciences, Franklin Lakes, NJ) for 30 min. The cells were incubated with fluorochrome-labeled mAb for 30 min at RT in the dark then were washed twice with 3 mL flow cytometry buffer (PBS + 0.5% BSA + 0.1% NaN_3_). Cells were post-fixed with 200 μL IC Fixation buffer (Life Technologies Corp.) for 30 min at RT in the dark, then washed again with 3 mL flow cytometry buffer, and stored at 4 °C until analysis. Flow cytometry was performed on a Stratedigm S1200Ex (Stratedigm Inc., San Jose, CA) and data were analyzed with CellCapTure software (Stratedigm).

### Intracellular cytokine staining

Cells (1 × 10^6^ cells/200 μL) were incubated overnight at 37 °C in 96-well plates containing DMEM/F12 + Glutamax (Thermo Fisher Scientific, Waltham, MA) containing 5% horse serum, 10% FBS and 1% Penicillin-Streptomycin. The following day, 2 μL of Cell Stimulation + Protein Transport Inhibitors (eBioscience, Inc., San Diego, CA) per 1 mL of solution was added to each well and cells were incubated for another 4 hrs. Cells were collected by adding EDTA to a final concentration of 2 mM, and transferred into 5 mL polystyrene tubes and washed twice with PBS containing 2 mM EDTA and 0.5% BSA. Cells were incubated on ice for 30 min with TruStain fcX and Brilliant Stain buffer as above. Fluorochrome-labeled mAb directed against extracellular antigens were added and cells were incubated at RT in the dark for 30 min then washed with flow cytometry buffer and fixed as above. Fixed cells were re-suspended in 1x Permeabilization Buffer (eBioscience, Inc.) at RT in the dark for 20 min, then pelleted and fluorochrome-labeled mAb against TNF, IFN-γ, and Granzyme B were added and gently mixed. Cells were incubated at RT in the dark for 20 min, followed by a final wash in flow cytometry buffer. Cells were stored in the dark at 4 °C until analysis. Flow cytometry was performed as above.

### Analysis of gene expression in CD11b^+^ brain cells

Brains were harvested from uninfected young and aged mice, digested as described above, and CD11b^+^ brain cells were collected by magnetic sorting using CD11b (Microglia) beads (Miltenyi Biotec) according to the manufacturer’s protocol. Cells were lysed in Qiazol Lysis Reagent, and total RNA was extracted using the miRNeasy Micro Kit (Qiagen, Redwood City, CA) according to the manufacturer’s instructions. Expression of miR-155 was measured using TaqMan microRNA reverse transcription and Taqman miRNA assay kits (Life Technologies Corp.). Primers for miR-155 (assay ID 002571) and snoRNA-135 (assay ID 00123, both from Invitrogen, Carlsbad, CA) were used in standard TaqMan assays according to the manufacturer’s protocol. Expression of miR-155 was normalized to snoRNA-135 within the same sample to account for variation in sample loading.

mRNA expression analysis with the NanoString nCounter® Immunology Panel (Mouse) was performed according to manufacturer’s protocol (NanoString Technologies Inc., Seattle, WA), and nCounts were normalized to the geometric mean of the internal positive controls using nSolver™ Analysis Software (NanoString Technologies Inc.). Further analyses of gene expression to identify key molecular pathways and upstream mediators was performed using Ingenuity Pathways Analysis (IPA) (Qiagen, Redwood City, CA). Heat maps of differentially expressed genes between young and aged mice were prepared using Heatmapper (www.Heatmapper.ca) [36].

### Statistical analysis

Statistical analyses of data are given in the figure legends. Briefly, a two-tailed Student’s *t* test assuming equal variance was used to compare two groups, and for a non-parametric distribution, the Mann-Whitney *U* test was used. With both tests a *p* < 0.05 was considered significant. Survival curves were analyzed using a Mantel-Cox log-rank test, whereas changes in weight over time between groups was analyzed using 2-way ANOVA followed by Tukey’s multiple comparisons test. Statistical analysis of results in comparing the accumulation of bT_RM_ during aging was performed using one-way ANOVA without the assumption of equal variance (Brown-Forsythe and Welch’s ANOVA tests) followed by a post-hoc comparison using Dunnett’s T3 multiple comparisons test. Statistical analysis related to gene expression data was performed with unpaired t-tests using individual variance for each row (gene) followed by correction for multiple comparisons by calculating False Discovery Rates (FDR) using the two-stage step up (Benjamini, Krieger, and Yekutieli) method with the Prism 9 statistical suite (GraphPad Software, San Diego, CA). FDR of q < 0.05 was considered statistically significant.

## Results

### Aged mice have excess mortality after lm infection and high homeostatic numbers of CD8^+^ bT_RM_

**Fig. 1:**
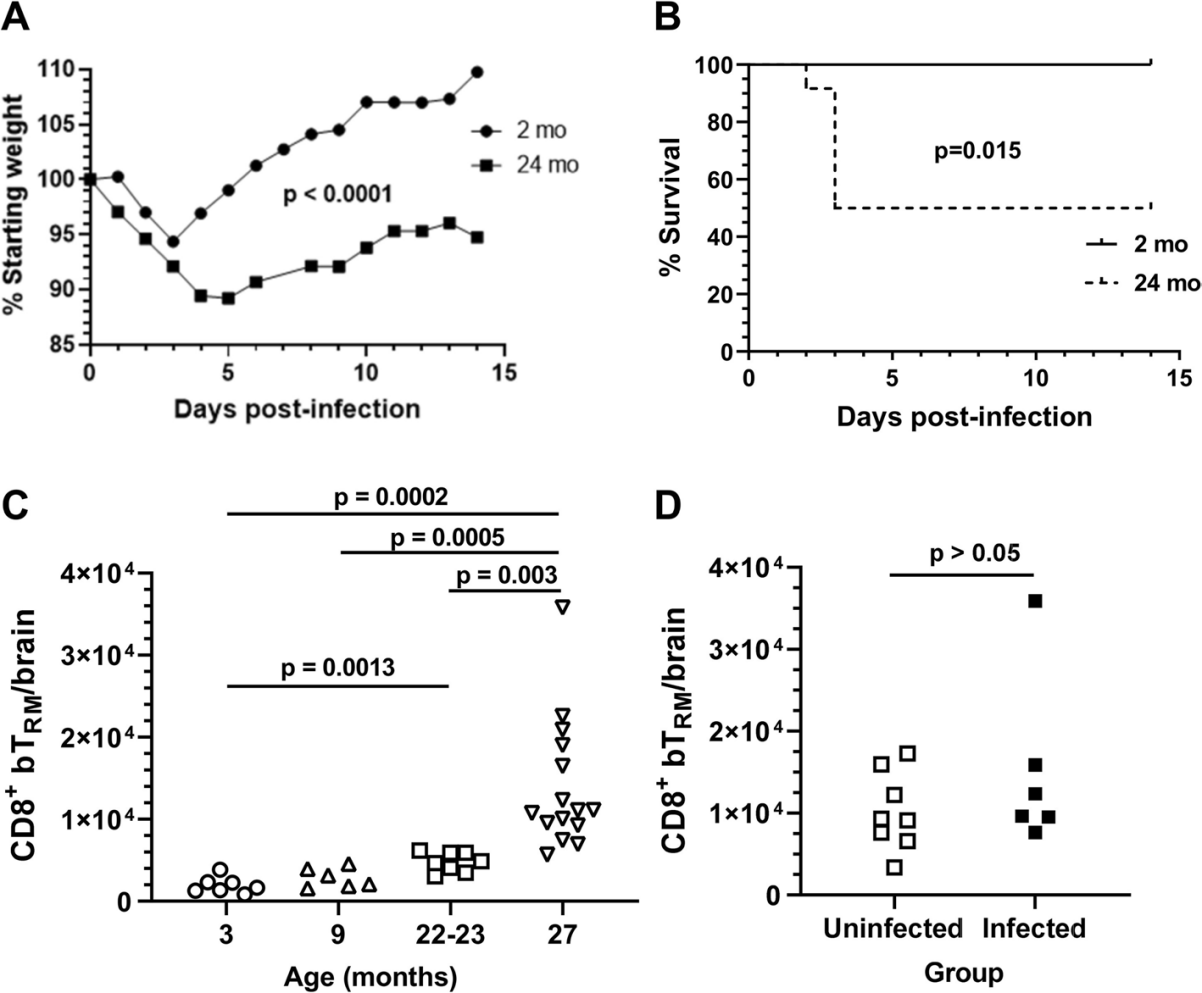
Young and aged mice differ in outcomes to *Lm* infection numbers of homeostatic CD8^+^ bT_RM_. 2 mo C57BL/6 J (●) and 24 mo C57BL/6JN (■) mice infected i.p. with *Lm* EGD 2.0 × 10^5^ CFU (*n* = 9) and 0.3–0.6 × 10^5^ CFU (*n* = 12), respectively (**A**, **B**, and **D**). All mice received antibiotics 2-16d p.i. Weight was measured daily and is shown as the mean % starting weight for surviving mice (**A**). Difference in weight loss between cohorts was calculated by 2-way ANOVA. Data in (**B**) show Kaplan-Meier survival plots of mice aged 2 mo (solid line) and 24 mo (broken line) after infection, significance calculated via log-rank test. Panels **C** and **D** show numbers of CD8^+^ bT_RM_ cells/brain in uninfected 3 mo C57BL6/J (Ο), 9 mo C57BL/6 N (△), 22–23 mo C57BL/6JN (□) and 27 mo C57BL/6JN (▽) male mice (**C**), and in 24mo old C57BL/6JN male mice 29d p.i. with 0.7 × 10^5^ CFU *Lm* (■) and uninfected (□) age-matched mice treated with antibiotics (**D**). Symbols represent individual animals. Statistical analysis by 1-way Brown-Forsythe and Welch ANOVA with Dunnett’s T3 multiple comparisons post-test (**C**) or by simple t-test (**D**)

To determine if *Lm* infection increases numbers of CD8^+^ bT_RM_ in aged mice, 24 mo old C57BL/6JN mice were infected with 0.3–0.7 × 10^5^ CFU *Lm* strain EGD whereas young mice were infected with 2.0 × 10^5^ CFU *Lm*, then both groups were treated with the same antibiotic protocol. The inoculum given to aged mice was reduced as prior experiments showed that aged mice were more susceptible to *Lm* infection than were young mice [37]. Although this is an essential difference between experimental groups, we deemed it a necessary solution when comparing infection-induced neuroinflammation in mice of such different ages. Despite the reduced inoculum, aged mice had significantly more weight loss after *Lm* infection than did young mice (*p* < 0.0001, 2-way ANOVA) (Fig. 1A). Maximum weight loss in young mice occurred at 3d p.i. then returned to pre-infection levels by 6d p.i. In contrast, aged mice did not return to their pre-infection weight over the course of observation. Aged mice also had significantly greater mortality than did young mice (*p* = 0.015 by log-rank test) (Fig. 1B), with only 6 out of 12 mice (50%) surviving to the end of observation, whereas there was no mortality among young mice. Although formal LD50 analyses were not done, prior studies using young C57BL/6 mice treated with antibiotics showed an 8% mortality rate among mice infected with a mean of 3.4 × 10^5^ CFU of this strain of *Lm* [6] and no mortality in mice when the inoculum was reduced to 2 × 10^5^ CFU [8]. Thus, the 50% mortality observed among aged mice after an approximate 3–6-fold reduction in inoculum despite antibiotic treatment compared with no mortality in young mice is striking.

Analysis of CD8^+^ bT_RM_ was accomplished by gating with standard markers as previously described [8] (Supplemental Fig. 1). In accord with prior studies, we found homeostatic numbers of CD8^+^ bT_RM_ increase during aging [16]. Results in Fig. 1C show statistically significant increases in numbers of CD8^+^ bT_RM_ in uninfected mice are detectable by 22–23 mo of age relative to 3 mo, with a nearly eightfold increase by 27 mo. Next, we tested the degree to which systemic wild-type *Lm* infection increased numbers of CD8^+^ bT_RM_ in aged mice as demonstrated before in young mice [8]. For a comparison with aged mice, 2 mo old C57BL/6 J mice were infected i.p. with a lethal dose of 2.0 × 10^5^ CFU *Lm* strain EGD, and rescued with antibiotics as described [6] (Fig. 1D). In contrast to our prior findings in young mice [6], numbers of CD8^+^ bT_RM_ measured 29d p.i. in the surviving aged mice were unchanged compared with uninfected mice (Fig. 1D). Thus, despite a 4-fold lower inoculum, aged mice lost significantly more weight and had greater mortality to *Lm* than did young mice. Nonetheless, aged mice had no apparent increase in numbers of CD8^+^ bT_RM_ after infection.

### Age-associated changes in gene expression and miR-155 targets in brain CD11b^+^ cells

**Fig. 2:**
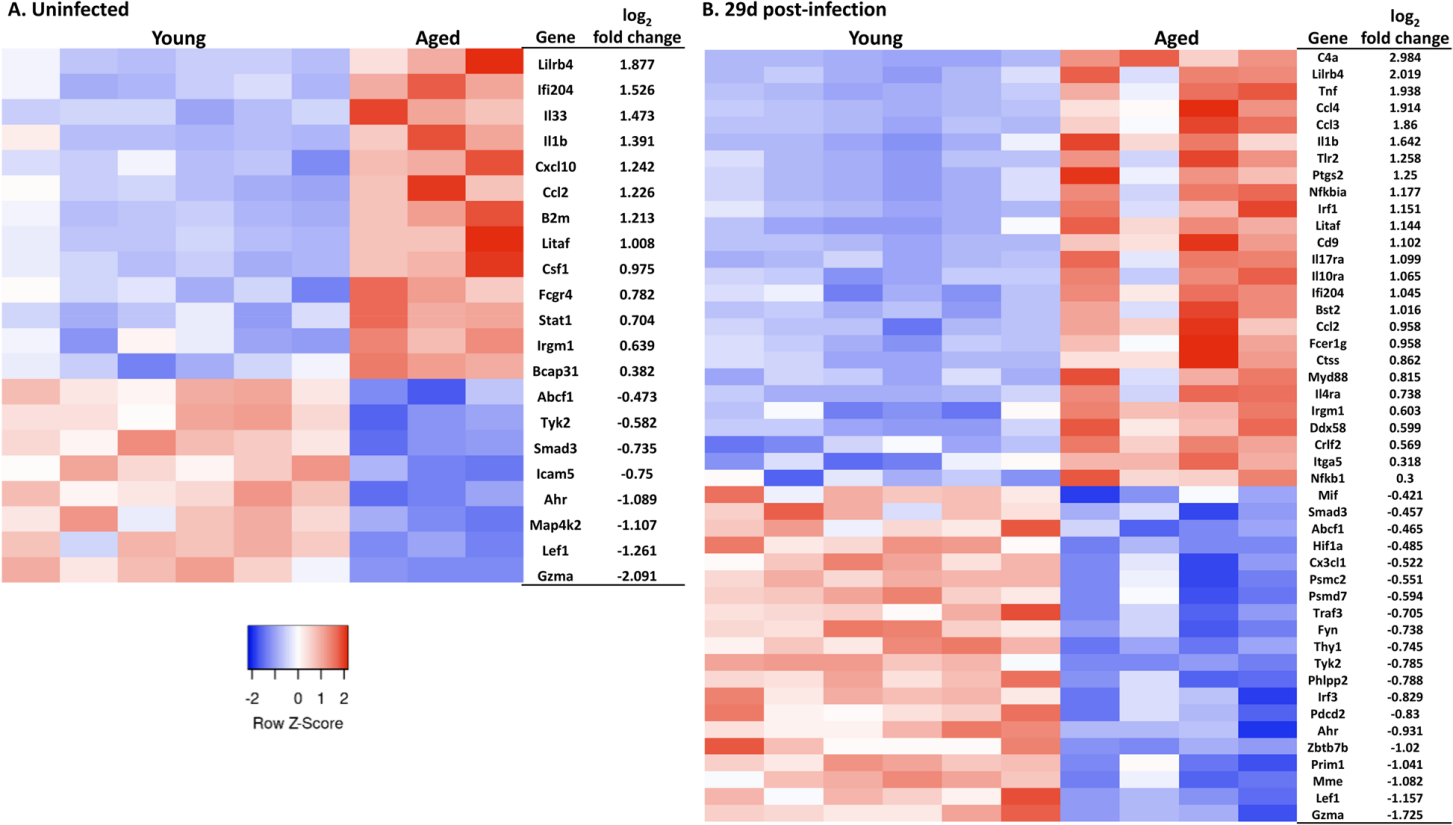
Aging alters homeostatic and post-infection gene expression in brain CD11b^+^ cells. CD11b^+^ brain cells were magnetically sorted from Young (2 mo) C57BL/6 J and Aged (24 mo) C57BL/6JN mice. Uninfected mice (**A**) were not manipulated. Infected mice (**B**) were injected i.p. with *Lm* EGD 2.0 × 10^5^ CFU (young) or 0.3–0.6 × 10^5^ CFU (aged), then received antibiotics 2d-16d p.i. and were euthanized 29d p.i. Gene expression was measured by nCounts. FDR of q < 0.05 was determined by multiple t-test followed by two-stage step up (Benjamini, Krieger, and Yekutieli) correction for multiple comparisons. Heat maps were based on the log_2_ fold change (aged/young) nCounts from significantly changed genes

**Fig. 3:**
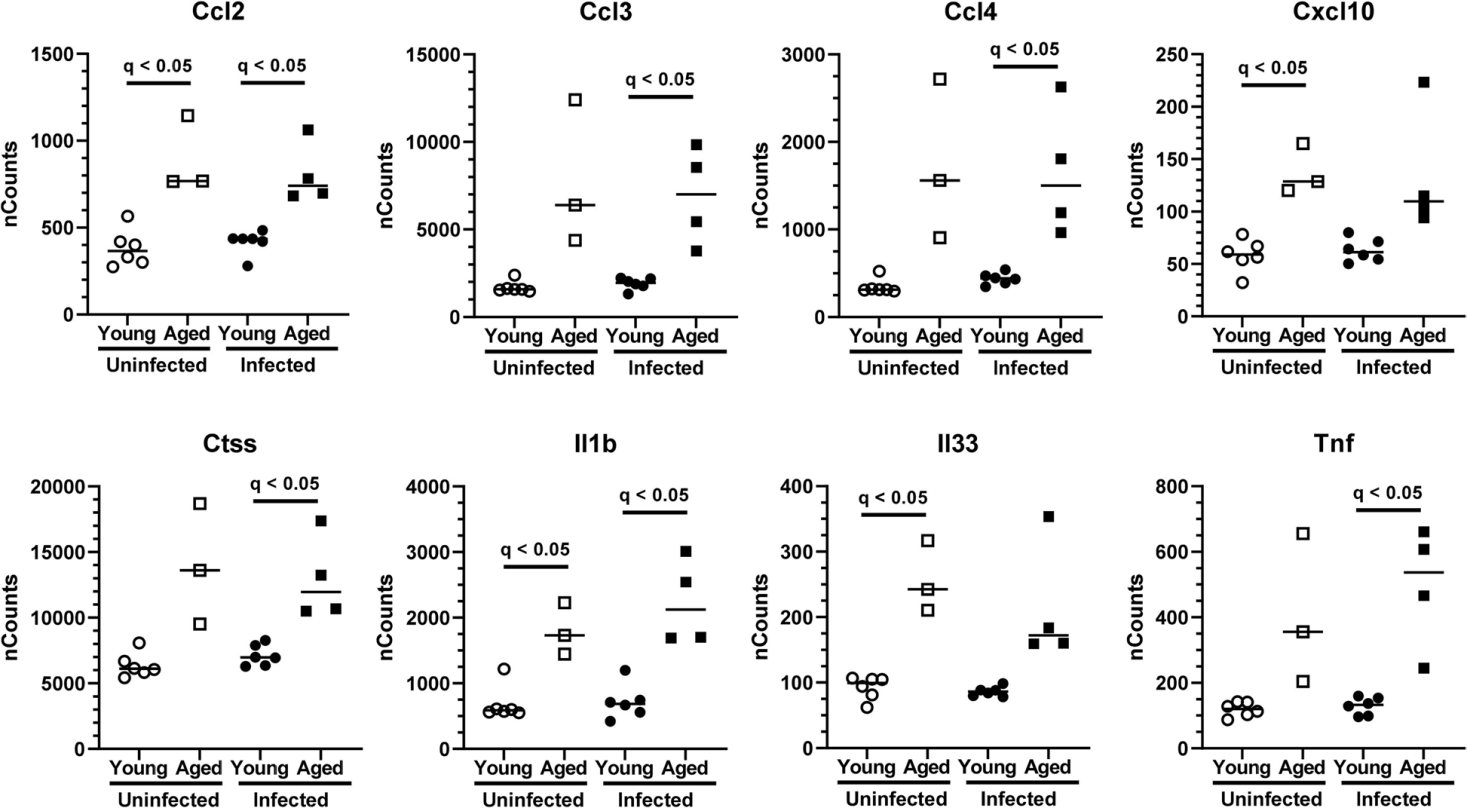
CD11b^+^ brain cells from aged mice display a pro-inflammatory signature. 2 mo C57BL/6 J and 24 mo C57BL/6JN mice were infected i.p. with *Lm* EGD 2.0 × 10^5^ CFU (*n* = 9) and 0.3–0.6 × 10^5^ CFU (*n* = 12), respectively, then received antibiotics beginning 2d p.i. CD11b^+^ brain cells were obtained from uninfected mice and infected mice 29d p.i. by magnetic sorting and gene expression was measured by nCounts. Statistical analysis was by multiple t-test followed by correction for multiple comparisons using the two-stage step up (Benjamini, Krieger, and Yekutieli) method. FDR of q < 0.05 was considered statistically significant

To identify aging-induced changes in neuroinflammatory responses to infection, as well as alterations that could contribute to differential recruitment of CD8^+^ bT_RM_ at steady state, we measured gene expression by nCounts using the NanoString nCounter® Mouse Immunology Panel in magnetically sorted CD11b^+^ brain cells from young and aged mice without infection, and 29d p.i.. Numbers of CD45^hi^CD11b^hi^ brain cells measured by flow cytometry pre-sort did not differ among any of the groups suggesting differential representation of these would not skew results (Supplemental Fig. 2) but were not re-studied post-sort. In uninfected mice, there 21 of 546 genes assessed had mRNA expression > 50 nCounts, i.e. copies/brain, in at least one group and which differed at a q < 0.05 (Fig. 2A, Supplemental Data File 1). These included pro-inflammatory, e.g. *Il1b*, and interferon-related genes, such as *Stat1, B2m,* and *Ifi204*, *Ccl2* and *Cxcl10,* the latter of which is important for memory CD8^+^ T-cell recruitment [38–40]. Comparison of gene expression in cells from aged and young mice 29d p.i. also revealed a pro-inflammatory pattern with 46 genes passing the same expression and statistical cutoffs (Fig. 2B). Genes of pro-inflammatory mediators generally had higher levels of expression in aged than in young mice (Fig. 3).

Analysis of upstream regulators showed PTGS1 (COX1) and Hepatocyte Growth Factor (HGF) were activated in in uninfected aged mice compared with young mice (Table 1), and both are associated with neuroinflammatory states [41, 42]. Moreover, inhibition of the repressor BCL6 also matches the pro-inflammatory condition associated with aging [43]. After infection, several additional pro-inflammatory upstream regulators including PTGS2 (COX2), IL1A, TNF, and SPI1 (PU.1) were activated at higher levels in aged mice (Table 1). The low number of samples and highly variable nCounts in aged mice, as well as possible survivorship bias, limited this analysis. Nonetheless, these results demonstrate that pre- and post-infection gene expression in brain CD11b^+^ cells from aged mice differs dramatically from young mice with cells from aged mice having a more pro-inflammatory gene signature. These differences, along with age-related changes in CD8^+^ T-lymphocyte chemokine receptor expression [44], could lead to age-related changes in accumulation of brain leukocytes at homeostasis as well as in response to infection. In addition, regulatory mechanisms in both groups of mice appear able to return most infection-induced changes in gene expression to their respective homeostatic levels.

**Table 1:** Analysis of upstream regulators and their predicted activation states in aged mice compared with young mice^a^

Comparison of aged vs young	Upstream Regulator	Predicted Activation State	Activation z-score	***p***-value
**Uninfected**	PTGS1	Activated	2.575	0.0316
	HGF	Activated	2.131	0.0015
	BCL6	Inhibited	−2.531	0.0011
**Infected**	IGF1	Activated	2.424	0.0279
	ITGB2	Activated	2.419	0.0212
	PTGS2	Activated	2.319	0.0419
	RAC1	Activated	2.247	0.0198
	SPI1	Activated	2.176	0.00028
	IL1A	Activated	2.038	0.000091
	TNF	Activated	2.004	0.0000015

^a^ Complete gene lists were analyzed in Ingenuity Pathways Analysis®. Results shown are upstream regulators with activation Z-scores ≥2.0 or ≤ −2.0 and a *p*-value of < 0.05. Statistical significance calculate via right-tailed Fisher’s Exact Test

**Fig. 4:**
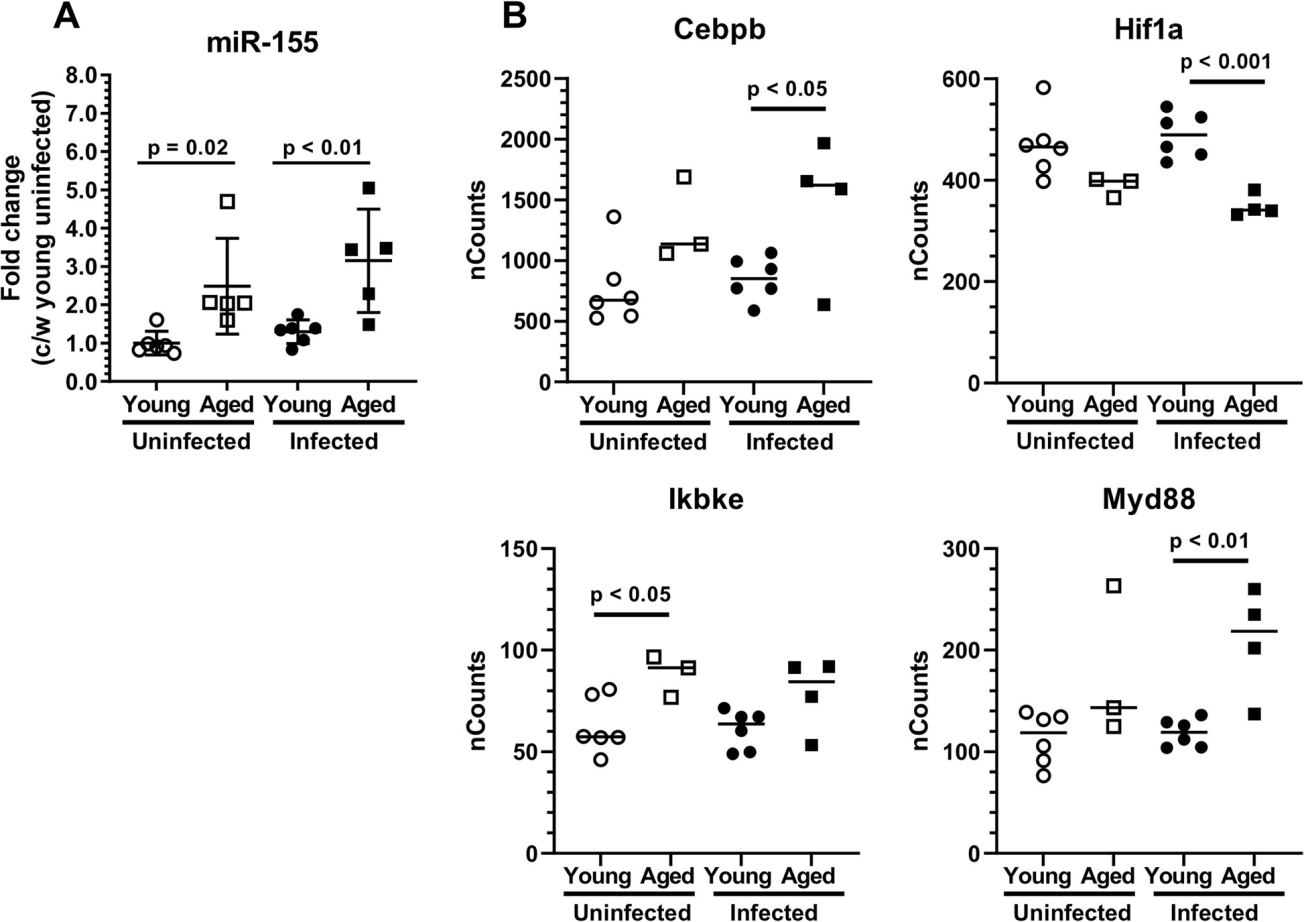
Differential expression of miR-155-targeted transcription factors in CD11b^+^ brain cells from young and aged mice. 2 mo C57BL/6 J and 24 mo C57BL/6JN mice were infected i.p. with *Lm* EGD 2.0 × 10^5^ CFU (*n* = 9) and 0.3–0.6 × 10^5^ CFU (*n* = 12), respectively, then received antibiotics beginning 2d p.i. CD11b^+^ brain cells were obtained from uninfected mice and infected mice 29d p.i. by magnetic sorting. Gene expression was measured by qPCR normalized to snoRNA-135 (A) or by nCounts (B). Statistical analysis was by simple t-test, a *p* < 0.05 was considered statistically significant

Expression of miR-155 was measured by qPCR in brain CD11b^+^ cells from young and aged mice (Fig. 4A). Cells from uninfected and infected aged animals had expressed significantly more miR-155 than did cells from uninfected and infected young animals, respectively. In addition, a review of all 547 genes measured on the NanoString panel identified 14 that are established targets of miR-155 [45] (Supplemental Fig. 3). Statistical analysis of this smaller list was performed by simple *t*-test and showed *Cebpb*, *Hif1a*, *Ikbke*, and *Myd88* were differentially expressed in young and aged mice (Fig. 4B). These data indicate that CD11b^+^ brain cells from aged mice, mostly comprised of microglia, have a significantly up-regulated inflammatory profile compared to those from young mice. Additionally, differential expression of miR-155 and miR-155 targets between aged and young mice could cause these two groups to respond differently to inhibition of miR-155 during infection.

Despite these findings, the degree to which CD8^+^ bT_RM_, or other leukocyte populations in the brain, are or are not increased after *Lm* infection is not clear. A key possibility is that the infective dose of bacteria, which was reduced several-fold to compensate for the excess mortality of aged mice, was too low to induce neuroinflammation. A second consideration is that the loss of 50% of infected animals could have compromised accurate evaluation of brain leukocyte numbers should animals with greatest numbers of brain leukocytes coincidentally also be the ones that die before analysis. To circumvent these issues with pathogenic *Lm*, we evaluated well-studied *Lm* mutants for their ability to induce leukocyte recruitment to the brain in the absence of excessive mortality.

### Lm ΔactA infection triggers CD8^+^ bT_RM_ recruitment to the brain in young mice without incurring significant mortality

**Fig. 5:**
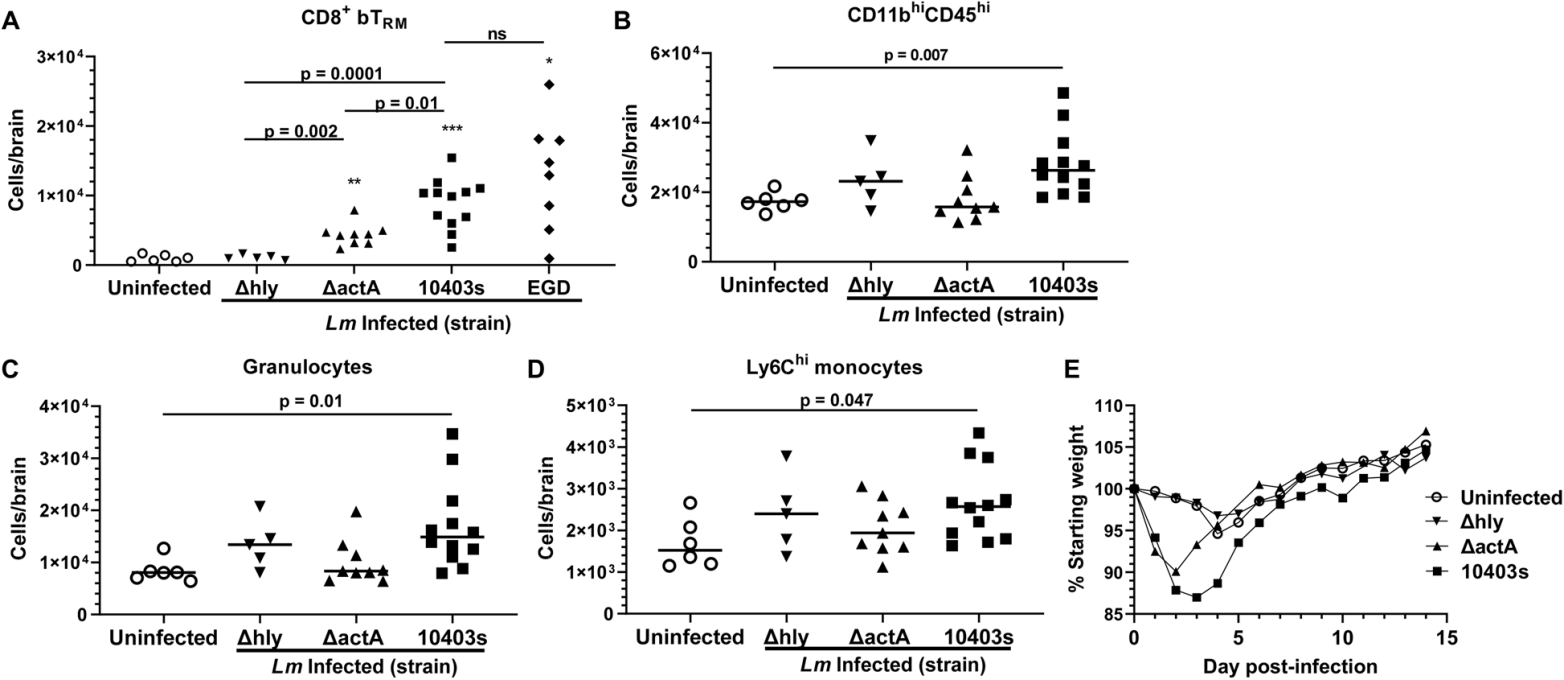
*Lm* mutants differentially trigger weight loss and accumulation of CD8^+^ bT_RM_ in young mice. 2 mo old C57BL/6 J mice were treated with antibiotics but not infected (○), or were infected with avirulent *Lm* mutants strain Δhly (▼, 1.6 × 10^7^ CFU), or ΔactA (▲, 2.0 × 10^7^ CFU), both from parent strain 10,403 s; or with wild type *Lm* strains 10,403 s (■, 3.2 × 10^6^ CFU), or EGD (♦, 1.2 × 10^5^ CFU). All mice were males with the exception that 4 M and 4 F mice were infected with EGD. **A-D** Symbols represent numbers of cells from individual mice harvested 29d-32d p.i. Significant differences between infected and uninfected mice are given as * *p* = 0.03, ** *p* = 0.001, and *** *p* < 0.0001. Significant differences between groups infected with different strains of *Lm* are shown. Significance was determined by 1-way Brown-Forsythe and Welch ANOVA with Dunnett’s T3 multiple comparisons post-test. Granulocytes in (**C**) were gated as CD11b^hi^CD45^hi^Ly6C^int^SSC^hi^, and represent mainly neutrophils but also include eosinophils. **E** Symbols represent mean percent of starting weight from 8 to 10 mice/group. Statistical significance calculated via 2-way ANOVA. Significance for selected comparisons: Uninfected v Δhly ns; Uninfected v. ΔactA *p* = 0.02; Uninfected v 10,403 s *p* < 0.0001; ΔactA v. 10,403 s *p* < 0.0001

Recent data show that systemic infection with *Lm* lacking the *actA* gene (Δ*actA*) triggers recruitment of peripherally induced CD8^+^ bT_RM_ to the brain [32]. This mutant is avirulent due to inability spread cell-to-cell after escaping phagosomes and could be a useful model for studying neuro-immune responses in aged mice. First, we compared accumulation of CD8^+^ bT_RM_ in young mice induced by Δ*actA Lm* infection with other *Lm* strains including Δ*hly Lm* mutants which neither escape phagosomes nor trigger cytosolic surveillance pathways (reviewed in [31]), the parent strain 10,403 s, and *Lm* strain EGD. All mice received antibiotics. Numbers of CD8^+^ bT_RM_ increased significantly in approximately 30d p.i. in mice infected with wild-type *Lm* strains 10,403 s and EGD (Fig. 5A). Infection with Δ*actA Lm* also triggered recruitment of CD8^+^ bT_RM,_ in young mice, albeit to a lesser degree than did strains 10,403 s and EGD. In contrast, numbers of CD8^+^ bT_RM_ were not changed from baseline in mice infected with Δ*hly Lm*. Analysis of bone marrow-derived myeloid cells showed significant recruitment to brains of 10,403 s-infected mice, but not in mice infected with Δ*actA* or Δ*hly Lm* (Fig. 5B-D). Mice infected with 10,403 s and Δ*actA* mutants had significant weight loss (Fig. 5E), although the magnitude was less in those infected with Δ*actA* mutants than with the parent strain. There were no mortalities among any infected mice. Next, a preliminary experiment performed in aged mice (21 mo) showed Δ*actA Lm* was cultured from spleen and liver, but not brain, 3d p.i. and that *Lm* Δ*actA* triggered brain influxes of activated CD8^+^ T-lymphocytes at 7d p.i. (Supplemental Fig. 4). Collectively these data support the notion of testing infection with Δ*actA Lm* mutants as a model for studying neuro-immune responses, particularly CD8^+^ bT_RM_, in aged mice.

### Systemic lm ΔactA infection triggers recruitment of cytokine-producing CD8^+^ bT_RM_ in aged mice without significant mortality

**Fig. 6:**
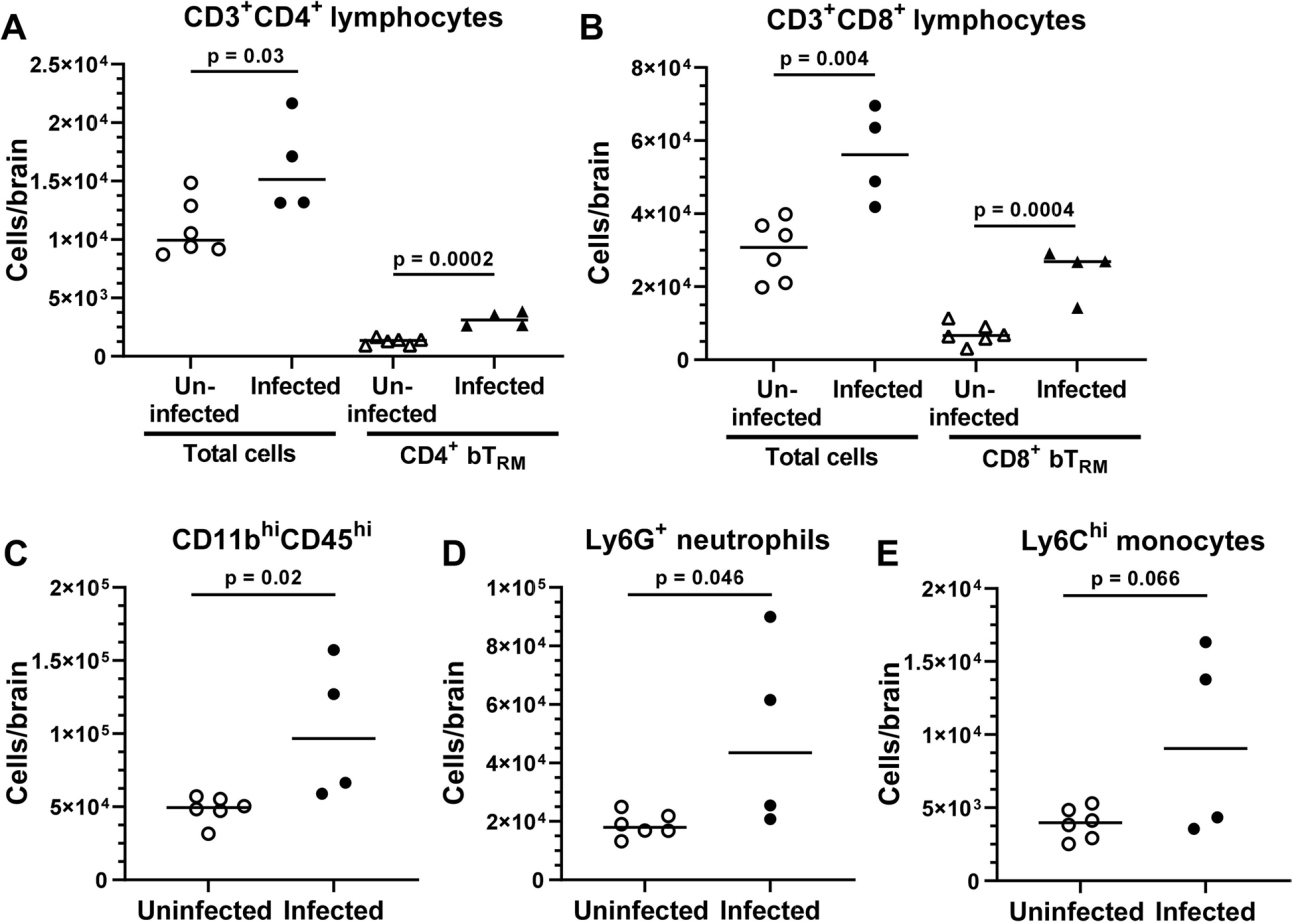
*Lm* ΔactA infection of aged mice triggers sustained leukocytosis in the brain. Male C57BL/6JN mice aged 19 mo were infected with 2.1 × 10^7^ CFU *Lm* ΔactA (*n* = 5, ●, ▲), or injected with PBS as uninfected controls (*n* = 6, ○, Δ). All mice received amoxicillin in their drinking water. One infected mouse died at 2d p.i. Mice were sacrificed 32d p.i., and brain leukocytes were analyzed by FACS. Symbols represent individual mice. Line indicates mean. Statistical significance calculated via Student’s *t* test

**Fig. 7:**
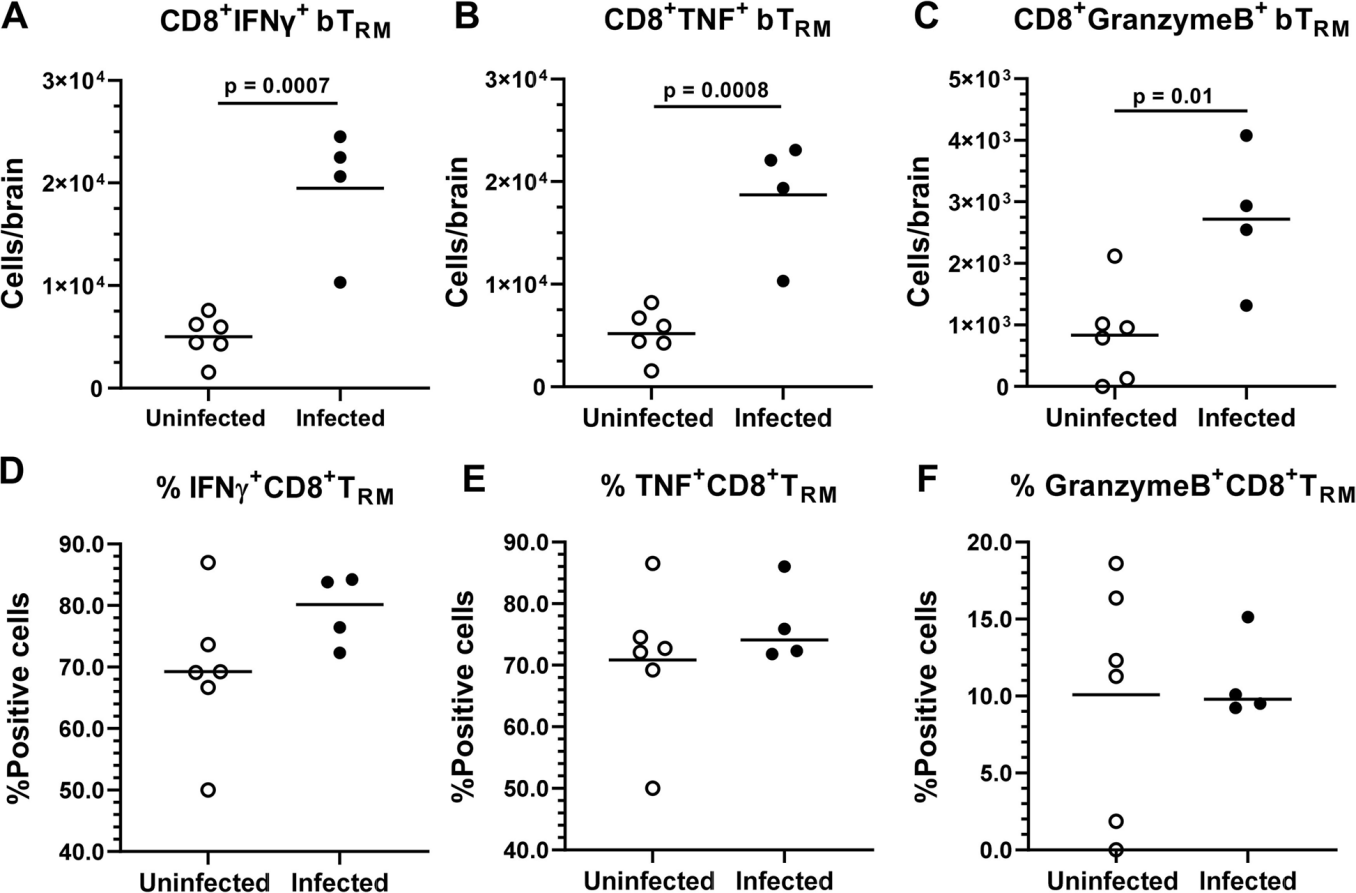
Infection of aged mice with *Lm* ΔactA increases numbers of cytokine-expressing CD8^+^ bT_RM_. Male C57BL/6JN mice aged 19 mo were infected with 2.1 × 10^7^ CFU *Lm* ΔactA (●), or injected with PBS as uninfected controls (○). All mice received amoxicillin in their drinking water and brain leukocytes were analyzed by FACS. Figs. (**A-C**) show results of intracellular staining of gated CD8^+^ bT_RM_ for IFNγ (**A**), TNF (**B**), and granzyme B (**C**). Symbols represent individual mice. Line indicates mean. Statistical significance calculated via Student’s *t* test

To study infection-induced CD8^+^ bT_RM_ in aging in more detail, 19 mo old mice were infected with *Lm* Δ*actA* or injected with PBS and given antibiotics. Of 5 infected mice, 1 died at 2d p.i. All mice were sacrificed 32d p.i. and brain leukocytes analyzed by flow cytometry. There were statistically significant increases in CD3^+^CD4^+^ and CD3^+^CD8^+^ lymphocytes upon infection (Fig. 6A, B). Importantly, CD4^+^ bT_RM_ (1300 ± 290 uninfected versus 3200 ± 610 infected (mean ± SD)) and CD8^+^ bT_RM_ (7100 ± 2820 uninfected versus 21,700 ± 6780 infected) were also significantly increased at 32d p.i. (Fig. 6A, B). In contrast to findings above in young mice infected with *Lm* Δ*actA* (Fig. 5B, C), numbers of total bone marrow-derived myeloid cells (CD45^hi^CD11b^+^) and Ly6G^+^ neutrophils were also increased 1 month after infection in two out of four mice (Fig. 6C, D), whereas the increase in Ly6C^hi^ monocytes, did not reach significance (Fig. 6E). Intracellular cytokine expression in CD8^+^ bT_RM_ was measured after in vitro stimulation with PMA/ionomycin using standard gating methods (Supplemental Fig. 5) showed total numbers of IFN-γ-, TNF-, and Granzyme B-expressing CD8^+^ bT_RM_ were significantly increased in infected mice compared to uninfected mice (Fig. 7 A-C). There were no statistically significant differences between infected and uninfected mice in the percentages of CD8^+^ bT_RM_ that expressed any of the mediators measured (Fig. 7 D-F). These results confirm that T_RM_ are indeed recruited increasingly to the brains of aged mice upon infection with Δ*actA Lm* and suggest that also myeloid cells show long-term brain recruitment in aged mice after infection.

### Inhibition of miR-155 suppresses recruitment of myeloid cells, but not CD8^+^ T_RM_, to brains of aged mice infected with lm ΔactA

**Fig. 8:**
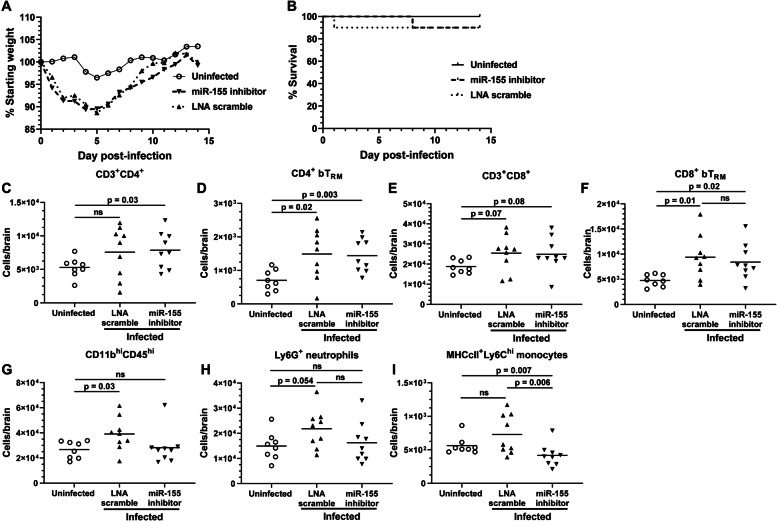
miR-155 inhibitor suppresses myeloid cell accumulation in brains of aged mice infected with *Lm* ΔactA. 22–23 mo old male C57BL/6JN mice were infected with 2.1 × 10^7^ CFU *Lm* ΔactA or injected with PBS as uninfected controls. All mice received amoxicillin in their drinking water beginning 3d p.i., and mice were given injections s.c. of miR-155 inhibitor (▼, *n* = 10), scramble (▲, *n* = 10), or PBS (○, *n* = 9) 2d, 4d, 6d, and 8d p.i. (**A**) Weight was measured daily and each symbol represents the mean % starting weight/cohort for that day. Statistical significance calculated via 2-way ANOVA. Uninfected v miR-155 inhibitor *p* < 0.0001; Uninfected v LNA scramble *p* < 0.0001; miR-155 inhibitor v LNA scramble ns. (**B**) Kaplan-Meier survival curves for each group. Brain leukocytes collected 29d – 38d p.i., were analyzed by FACS (C-I). Symbols represent individual mice, lines indicate group mean. *P* values shown were calculated with Student’s *t* test or Mann-Whitney *U* test

Our prior findings showed that injection of anti-miR-155 oligonucleotide into young mice infected systemically with *Lm* strain EGD decreased numbers of CD8^+^ bT_RM_ 28d p.i., whereas numbers of CD4^+^ bT_RM_ and myeloid cells were not changed [8]. Using the Δ*actA Lm* infection model, we could also test if anti-miR-155 also decreased recruitment of CD8^+^ bT_RM_ in aged mice. For this, aged mice were infected with *Lm* Δ*actA* and treated with oral amoxicillin as before. Infected mice were then injected s.c. with either anti-miR-155 oligonucleotide or control oligonucleotide (LNA scramble) 2d, 4d, 6d, and 8d p.i. Uninfected control mice were injected with PBS instead of *Lm*, then were injected s.c. with PBS rather than oligonucleotide. Weight was measured daily through 14d p.i. and mice were sacrificed 29d-38d p.i. to quantify brain leukocytes. Results in Fig. 8A show that both groups of infected mice lost significant weight compared to uninfected mice (*p* < 0.0001 for each, 2-way ANOVA), but there was no difference between anti-miR-155-treated mice and mice injected with the LNA scramble. Similarly, there were no differences in mortality with 1 death in each infected group (Fig. 8B).

**Fig. 9:**
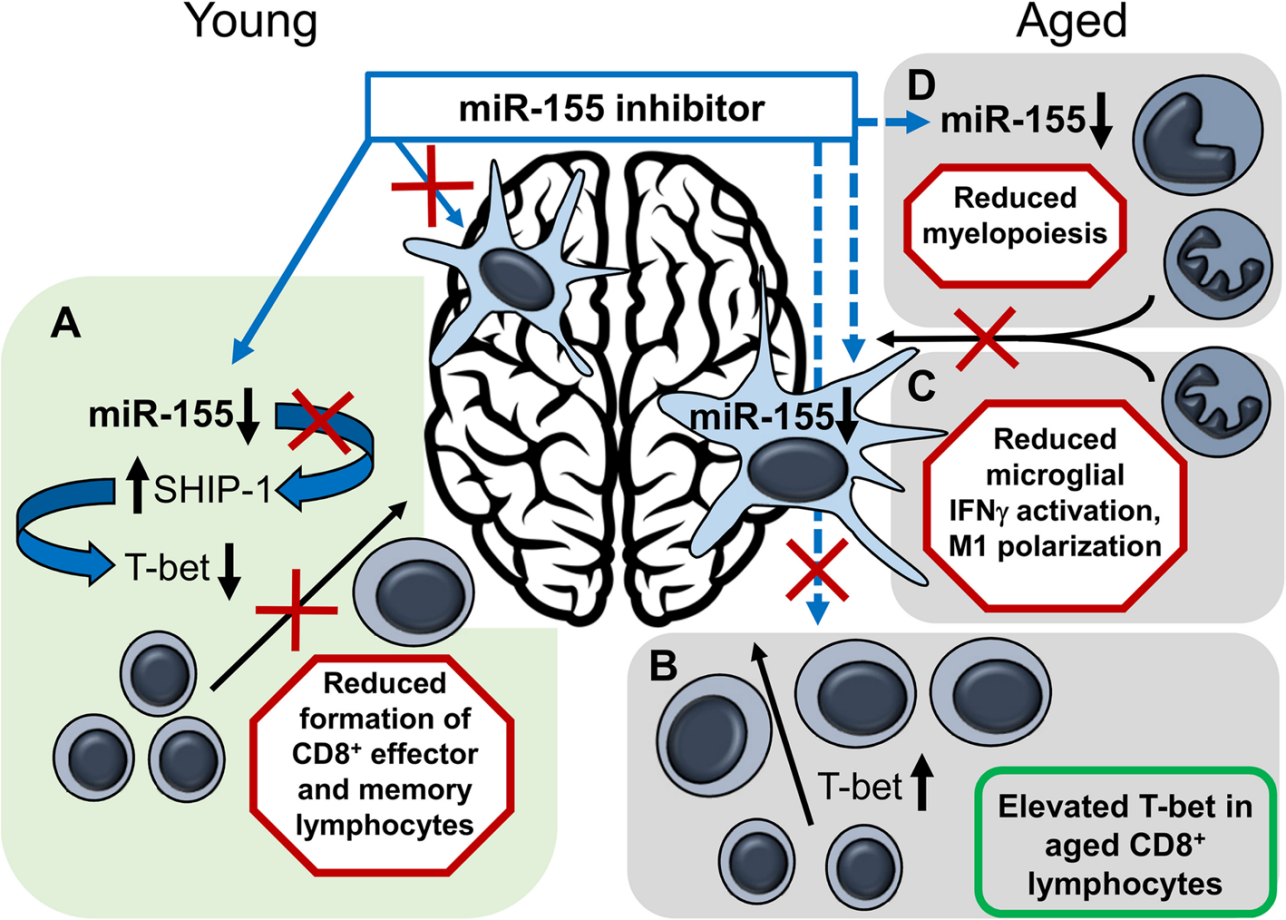
Proposed mechanisms underlying differential action of miR-155 inhibition in young and aged mice. In young mice, inhibition of miR-155 leads to reduced formation of effector and memory CD8^+^ T-lymphocytes via downregulation of T-bet, whereas it has no measurable effect on microglia (**A**). In aged mice, reduced numbers of naïve CD8^+^ T-lymphocytes and elevated basal T-bet expression combine to obscure this effect of miR-155 (**B**). miR-155 inhibitor may cross the blood-brain barriers in aged mice and there inhibit infection-induced IFNγ-activation and M1-polarizaiton of microglia thereby limiting production of chemoattractants for recruiting leukocytes into the brain (**C**). Aging is accompanied by a shift of the immune response towards myelopoiesis, rendering these cells more susceptible to inhibition of miR-155 (**D**). Proposed mechanisms in **C** and **D** both result in reduced myeloid cell accumulation in the brain in mice treated with miR-155 inhibitor. Solid blue arrows represent the effect of miR-155 inhibitor in young mice, proposed effects of miR-155 in aged mice are represented by hatched blue arrows. Black arrows show the net effects of miR-155 inhibitor on leukocyte accumulation in the brain

In agreement with results above, CD4^+^ bT_RM_ and CD8^+^ bT_RM_ numbers were increased after infection with *Lm* Δ*actA* (Fig. 8 D, F). However, contrary to our previous findings in young mice, anti-miR-155 caused no significant reduction of CD8^+^ bT_RM_ numbers or other populations of lymphocytes (Fig. 8C-F). In contrast, numbers of myeloid cells (CD11b^hi^CD45^hi^), which were increased in mice treated with LNA scramble compared to uninfected mice (*p* = 0.03), were at the same level in anti-miR-155-treated mice as in uninfected controls (Fig. 8G). Further analysis showed this was largely ascribed to increased numbers of Ly6G^+^ neutrophils in LNA scramble-treated mice (*p* = 0.054, Fig. 8H). Additionally, MHCII^+^Ly6C^hi^-expressing monocytes were decreased in anti-miR-155-treated mice (*p* = 0.006) compared to LNA scramble-treated mice, and even lower when compared to uninfected mice (*p* = 0.007, Fig. 8I). Thus, instead of specifically inhibiting accumulation of CD8^+^ bT_RM_ as in young mice, inhibition of miR-155 suppressed late accumulation of myeloid cells in the brains of aged mice. Figure 9 gives a comparison of the the effect of anti-miR-155 treatment on numbers of CD8^+^ bT_RM_ and bone marrow-derived myeloid cells in the brains of young and aged mice from experiments reported here, and in our prior study [8].

## Discussion

Brain inflammation is a key cause of poor neurological outcomes after severe infections such as meningitis and sepsis, particularly in the elderly [1, 2]. However, models to study treatment of severe infections in aged animals can be challenging as aged mice have higher mortality than do young mice in some models of sepsis and meningitis [37, 46–48]. Nonetheless, it is critically important to study severe infections in aged mice in order to identify cellular and molecular changes induced by aging that impact host responses [49, 50].

miR-155 is a short, non-coding RNA expressed in a wide variety of cells during inflammation and immune activation [51]. It is an important regulator of neuroinflammation in diverse pathological conditions e.g. multiple sclerosis and Alzheimer’s disease, and blockade of miR-155 can improve some features of key disease processes suggesting it could be an important therapeutic target [52–54]. In addition to having a role in age-related neurodegenerative diseases, overexpression of miR-155 in T-lymphocytes reduces the lifespan of miR-146a^−/−^ mice in a model of chronic inflammation, and recent studies show that aged miR-155^−/−^ mice have less spontaneous pain and decreased mortality after experimental spinal cord injury than do normal mice [55, 56]. In an model of antibiotic treatment of neuroinvasive *Lm* infection, we showed that miR-155 has multiple roles, the most prominent of which is being required for optimal development of IFN-γ-producing CD8^+^ T-lymphocytes that activate microglia and promote M1 polarization of microglia, although it also can inhibit microglial production of pro-inflammatory mediators [6]. Notably, inhibition/deletion of miR-155 reduces brain accumulation of CD8^+^ T_RM_ after *Lm* infection [8]. Yang et al. also found that miR-155 also regulates microglial inflammatory responses by providing negative feedback regulation of TLR-mediated NF-κB and EGFR-NF-κB signaling pathways during acute *E. coli* meningitis in 4 wk. old mice [57]. In contrast to these data, post-infectious brain inflammation in aged mice and the role of miR-155 in this setting not been studied.

Based on prior results in young mice, we studied accumulation of infection-induced CD8^+^ bT_RM_ in aged mice and the putative involvement of miR-155 in this process [8]. Initial results with wild-type *Lm* showed that aged mice (24 months old) had significantly higher mortality and greater weight loss after infection than did younger mice, even when the inoculum was reduced by 4-fold. Notably, increasing age also contributes significantly to higher mortality in human listeriosis supporting the clinical relevance of this model [22, 26]. Analysis of gene expression in resident brain CD11b^+^ cells showed cells from aged mice have a pro-inflammatory signature compared with those from younger mice, in accordance with previous studies (e.g. [58]). Interestingly, gene nCounts had essentially returned to their respective pre-infection levels in both young and aged mice by 29d p.i. There was a suggestion that expression levels of *Il1b* and *Tnf* were increased after infection in aged mice compared to uninfected mice, but the differences were not statistically significant. However, IPA identified pro-inflammatory upstream regulators including PTGS2 (COX2), IL1A, and TNF that were activated after infection in aged mice.

Analysis of gene expression in CD11b^+^ brain cells after *Lm* infection also identified significant differences between aged and young mice in expression of miR-155 targets. In particular, altered expressions of *Cebpb* and *PU.1* during aging and infection could have major impacts on cells recruiting into the CNS and are discussed in detail below. Together, these data suggest that inhibition of miR-155 could target different molecules in aged and young mice, therefore differentially modulating their immune responses to infection. Nevertheless, the 50% mortality of aged mice following *Lm* infection could have created an artifact due to survivorship bias. Thus, it was unclear whether unchanged numbers of CD8^+^ bT_RM_ post-infection reflected real differences between aged and young mice in the biology of neuroinflammation and CD8^+^ bT_RM_. Furthermore, observed gene expression changes are also likely an underestimation, as non-survivors would have a more pronounced neuroinflammatory profile.

A key advantage of the *Lm* infection model is the existence of well-characterized *Lm* mutants that can be used to probe immune responses. *Lm* Δ*hly* and *Lm* Δ*actA* mutants were used for in vivo infection and results compared with the wild-type parent strain *Lm* 10,403 s. Both mutants are highly attenuated due to the abrogation of cell-to-cell spread resulting from the inability to escape phagosomes (Δ*hly*) or to the loss of intracellular motility (Δ*actA*) [59, 60]. However, the intracellular lifecycles of *Lm* Δ*actA* and Δ*hly* mutants differ in critical and relevant ways. Specifically, Δ*actA*, but not Δ*hly* mutants, secrete listeriolysin O, escape phagosomes, and replicate within the cytosol of infected cells triggering the cGAS-STING cytosolic DNA sensing pathway (reviewed in [31]). This leads to production of IL-12, IL-18, and type I and type II IFNs. A critical result of these events is that systemic infection with *Lm* Δ*actA* mutants, but not Δ*hly* mutants, trigger brain influxes of Ly-6C^hi^ monocytes and activated CD8^+^ T-lymphocytes by 2d and 7d after infection, respectively, in the absence of brain infection [6, 61]. Additionally, Urban et al. showed that Δ*actA Lm* mutants induce brain recruitment of CD8^+^ T_RM_ that are phenotypically and functionally identical to brain CD8^+^ T_RM_ that follow neurotropic *Lm* infection [32]. Thus, infection with Δ*actA Lm* mutants is likely to be a useful model for studying infection-induced accumulation of CD8^+^ bT_RM_ in aged mice.

Infection with Δ*actA Lm* mutants caused only 3 deaths in 25 aged mice across all experimental groups, compared with 6 deaths of 12 aged mice infected with wild type *Lm*. Thus, excessively high mortality and the possibility of survivorship bias were avoided in aged mice. This strategy allowed careful investigation of key differences between young and old mice in the immune responses to the same inoculum of bacteria. We found that *Lm* Δ*actA* infection induced recruitment of CD8^+^ and CD4^+^ bT_RM_ in aged mice, along with significantly greater numbers of cytokine-expressing CD8^+^ bT_RM_ compared to uninfected mice. Additionally, there were increased numbers of neutrophils 29-32d p.i. following infection with Δ*actA* whereas this finding is not observed in young mice. These results show that systemic infection in aged mice, even in the absence of bacterial neuroinvasion, can induce long-term alterations in the neuroinflammatory environment. Compared with young mice, the finding of increased numbers of myeloid cells in brains of aged mice after systemic, non-neuro-invasive infection is likely a manifestation of the senescence-associated secretory phenotype, and was also observed in the heightened pro-inflammatory phenotype of aged microglia after infection [62]. Although it is possible that persistent myeloid cells could contribute to post-infectious neuroinflammation, their physiological relevance has not yet been established and they should be viewed as one component of a broader phenotype. Nonetheless, the finding of persistent, post-infectious neuroinflammation after non-neuroinvasive infection has wide-ranging implications. For example, it suggests other infections, particularly those caused by intracellular pathogens that trigger production of similar cytokines or a cytokine storm, e.g. severe influenza or SARS-CoV-2, could also drive brain influxes of CD8^+^ bT_RM_ as well as other leukocytes. The resultant neuroinflammation could drive cognitive dysfunction and would be especially detrimental in the elderly as they are less able to compensate for neuro-immune perturbations [63–67].

The inflammation-related microRNA miR-155 has important roles in neuroinflammation caused by diverse CNS insults. It is essential for optimal CD8^+^ T-lymphocyte responses to bacterial and viral infection [68–71]. Conversely, inhibition of miR-155 is protective in models of noninfectious inflammatory process such as stroke and experimental autoimmune encephalitis [72–74]. Our prior work in young adult animals showed reduced accumulation of CD8^+^ bT_RM_ following neuroinvasive *Lm* infection in mutated mice lacking miR-155, as well as in mice injected with an anti-miR-155 oligonucleotides [8]. Thus, we tested the extent to which anti-miR-155 also inhibited CD8^+^ bT_RM_ accumulation in aged mice infected with Δ*actA Lm* mutants. In contrast to findings in young mice, results presented here show that inhibition of miR-155 in aged mice did not change the accumulation of these cells. Instead, there was a significant decrease in brain accumulation of bone-marrow derived myeloid cells, including Ly6G^+^ neutrophils and MHCII^+^Ly6C^hi^ monocytes (Fig. 8). Although the physiologic significance of the absolute differences in these cell populations is not known, the differential trends in these data suggest that miR-155 exerts different effects in the brain immune response in aged mice compared to young mice.

Expression and activity of miR-155 are highly cell type- and context-dependent [75]. In general, miR-155 expression increases with aging in BM-derived macrophages, but is down-regulated in aging of vascular cells and peripheral blood mononuclear cells [76–78]. The putative mechanism by which miR-155-inhibitor reduces CD8^+^ bT_RM_ in young mice is by antagonizing the effect of miR-155 on its direct target SH2 (Src homology 2)-containing inositol phosphatase-1 (SHIP-1) that acts upon T-bet, a transcription factor required for CD8^+^ effector memory cell formation [79]. Specifically, inhibiting miR-155 expression during infection-induced expansion of CD8^+^ T-lymphocytes increases expression of SHIP1 that then down-regulates T-bet levels, therefore reducing formation of memory and effector CD8^+^ T-lymphocytes (Fig. 9A). Notably, T-bet expression in CD8^+^ T-lymphocytes increases with age and chronic activation [80]. This may render the effect of anti-miR-155 oligonucleotides ineffective for dampening T-bet expression in cells of aged mice (Fig. 9B). In addition, fewer naïve CD8^+^ T-cells in aged mice may be available to be targeted by miR-155 inhibitor in aged mice as they have reduced expansion of antigen-specific CD8^+^ T-lymphocytes during *Lm* infection compared with young mice [81].

The mechanisms causing reduced brain influxes of myeloid cell in aged mice treated with miR-155 inhibitor could originate in the brain or bone marrow. Our prior study in young mice showed miR-155 inhibitor caused no changes in microglial gene expression [8]. The age-related breakdown in the blood-brain barriers could allow resident brain cells such as microglia access to the inhibitor [82]. In this case, inhibition of microglial miR-155 during infection could lead to reduced production of chemoattractants for and hence less recruitment to the brain (Fig. 9C) [6]. Since microglia in aged mice display an increased inflammatory profile and higher miR-155 expression than in young mice, they could be more affected by miR-155 inhibition resulting in decreased expression of TNFα and chemokines e.g. CCL2 [83]. However, it seems more likely that age-related changes in hematopoiesis and expression of miR-155-targeted transcription factors underlie the finding of reduced numbers of neutrophils and monocytes in brains of aged mice treated with anti-miR-155 oligonucleotides. Aging is accompanied by a multi-faceted shift favoring myelopoiesis over lymphopoiesis [84]. Thus, the role of miR-155 driving myelopoiesis during acute inflammation, e.g. after lipopolysaccharide (LPS) injection, could be more pronounced and prolonged in aged mice than in young mice and therefore more susceptible to miR-155 inhibition (Fig. 9D) [85]. This suggests an increasing contribution of miR-155 to inflammation in aged mice in general. However, complexity of the involved networks is illustrated by the notion that chronic inflammation associated with aging is supported by increased mRNA and protein levels of the myeloid transcription factor SPI1/PU.1 [86] that is directly targeted by miR-155 [87–89]. Reducing miR-155 expression via an inhibitor would increase PU.1 expression and favor monopoiesis over granulopoiesis [87]. Interestingly, antagonism of miR-155 also down-regulates granulocyte colony-stimulating factor (G-CSF) expression in splenocytes of LPS-treated mice via de-repression of the transcription factor CCAAT/enhancer binding protein-β (C/EBPβ) [90]. Such a reduction of G-CSF could more profoundly impair granulopoiesis in aged mice than in young mice, or prolonged up-regulation of C/EBPβ in inhibitor-treated aged mice could interrupt the switch from C/EBPβ-controlled emergency granulopoiesis to C/EBPα-controlled steady-state granulopoiesis [91].

Limitations to these data include that results using miR-155 inhibitor on cellular infiltration to the brains of *Lm*-infected young and aged mice were derived using different strains of bacteria. Specifically, prior work in young mice used wild type *Lm* (strain EGD) that causes CNS infection [8], whereas the present study in aged mice used attenuated Δ*actA Lm* mutants. Thus, it is possible that different experimental results were due to the bacteria used rather than the age of mice. Nonetheless, our prior work in young mice shows that brain recruitment of Ly6C^hi^ monocytes by these different strains of *Lm* both depends upon IFNγ-dependent mechanisms [61, 92]. Moreover, peripherally-induced CD8^+^ bT_RM_ are highly similar to, if not identical to bT_RM_ generated by CNS infection suggesting their recruitment are also similar to each other [32]. Additionally, effects of miR-155 inhibitor on gene expression were not investigated in different tissues, e.g. brain, spleen, and bone marrow. Such data could help clarify mechanisms of differential responses in young and aged mice.

## Conclusions

The model presented here of *Lm* infection and treatment using aged mice is physiologically relevant in that it recapitulates a key feature of listeriosis in elderly humans, namely a significantly higher morbidity and mortality rate than seen in young mice. Using this model, we found significant differences in gene expression of CD11b^+^ brain cells between aged and young mice after *Lm* infection. These differences underscore that age-induced differences in mortality are reflective of significant differences at other levels, and also can impact responses of aged individuals to immune modulation. Systemic infection with *Lm* Δ*actA* mutants is a novel model for studying infection-induced brain inflammation in aged mice without excessive mortality. In this system we found that numbers of CD8^+^ bT_RM_ increase in both young and aged mice after infection, whereas bone marrow-derived myeloid cells increase only in aged mice. In contrast to the earlier findings in young mice, anti-miR-155 did not inhibit brain accumulation of CD8^+^ bT_RM_ in aged mice, but did suppress myeloid cell accrual. These results show young and aged mice differ in manifestations and mechanisms of infection-induced neuroinflammation and give insight for developing therapies to ameliorate brain inflammation following severe infection in the elderly. Specifically, they provide clear evidence that the role of miR-155 in response to bacterial infection changes with age. Multiple age-induced changes including expression of miR-155 and miR-155 targets, greater abundance of T-bet, and less robust expansion of CD8^+^ T-lymphocytes in aged mice likely contributed to the failure of anti-miR-155 oligonucleotides to inhibit brain accumulation of CD8^+^ bT_RM_ in aged mice. Nonetheless, because myeloid cells, in particular neutrophils, are key contributors to infection-induced neuroinflammation, reducing their accumulation in the brain is likely to be beneficial to long term neuro-cognitive outcomes [5].

## Supplementary Information

**Additional file 1: Supplemental Fig. 1.** Gating of brain CD8^+^ T_RM_. Representative FACS gating for CD8^+^ bT_RM_. Mouse shown was an uninfected 22 mo C57BL/6JN mouse.

**Additional file 2: Supplemental Fig. 2.** Young and aged mice have similar numbers of CD11b^hi^CD45^hi^ brain cells before and after infection. 2 mo C57BL/6 J and 24 mo C57BL/6JN mice were infected i.p. with *Lm* EGD 2.0 × 10^5^ CFU (*n* = 9) and 0.3–0.6 × 10^5^ CFU (*n* = 12), respectively, then received antibiotics beginning 2d p.i. Brain leukocytes were obtained from uninfected mice and from infected mice 29d p.i. by enzymatic digestion then were incubated with mAb and analyzed by flow cytometry. Symbols represent individual young uninfected (○), young infected (●), aged uninfected (□) and aged infected (■) mice. Line indicates mean. Statistical significance calculated via Student’s *t* test.

**Additional file 3: Supplemental Fig. 3.** Transcription factors targeted by miR-155 measured in CD11b^+^ brain cells from young and aged mice. 2 mo C57BL/6 J and 24 mo C57BL/6JN mice were infected i.p. with *Lm* EGD 2.0 × 10^5^ CFU (*n* = 9) and 0.3–0.6 × 10^5^ CFU (*n* = 12), respectively, then received antibiotics beginning 2d p.i. CD11b^+^ brain cells were obtained from uninfected mice and infected mice 29d p.i. by magnetic sorting and gene expression was measured by nCounts. Statistical analysis of this restricted list of genes was by simple t-test, a *p* < 0.05 was considered statistically significant.

**Additional file 4: Supplemental Fig. 4.** Preliminary studies of ΔactA *Lm* infection in aged mice. Panel 20 mo old C57BL/6 J mice (3F, 2 M) were infected with 2.0 × 10^7^ CFU *Lm* Δ*actA* (A). Mice were sacrificed D3 p.i., organs were harvested aseptically, homogenized in sterile dH_2_O, and bacterial CFU were measured by serial dilution and plating on agar. 21 mo C57BL/6JN male mice were infected i.p. with 3 × 10^7^ CFU *Lm* Δ*actA* or remained uninfected and given antibiotics starting 2d p.i. (B-E). Mice were harvested 7d p.i. Representative FACS dotplots (20,000 cells/plot) of gated CD45^+^ cells from uninfected (B) and infected (C) mice at 7d p.i. are shown. Myeloid cells are characterized by CD11b expression, while lymphocytes are essentially CD11b-negative. Data show numbers of brain lymphocytes (D) and myeloid cells (E) cells from individual infected (●) and uninfected (○) mice. Statistical significance between groups calculated by Mann-Whitney *U* test.

**Additional file 5: Supplemental Fig. 5.** Gating for intracellular cytokine analysis. Representative FACS dotplots of intracellular cytokine staining as shown in Fig. 6. Dotplots show selection of CD8^+^ T-lymphocytes from gated brain CD11b^neg-low^45^hi^CD3^+^ cells (A) incubated with anti-IFNg mAb (B) or isotype control (C), which was used to establish expression of IFNγ. Panels (D-G) display similar dotplots for Granzyme B and TNF expression.

**Additional file 6.**

## Data Availability

Data are available upon reasonable request.

## Abbreviations

BM: bone marrow
bT_RM_: Brain T_RM_
C/EBP: CCAAT/enhancer binding protein
CNS: central nervous system
d: days
FDR: false discovery rate
G-CSF: granulocyte colony-stimulating factor
hrs: hours
i.p.: intraperitoneal
i.v.: intravenous
IPA: Ingenuity Pathways Analysis
LPS: lipopolysaccharide
*Lm*: *Listeria monocytogenes*
LNA: Locked nucleic acid
min: minutes
mo: months
p.i.: post infection
RT: room temperature
s.c.: subcutaneous
T_RM_: Tissue-resident memory T cell
