# Supplementary figures and images for: Systemic Listeria monocytogenes infection in aged mice induces long-term neuroinflammation: the role of miR-155
Source: Immun Ageing. 2022 May 25;19:25. doi: 10.1186/s12979-022-00281-0 (PMC9130456; doi:10.1186/s12979-022-00281-0)

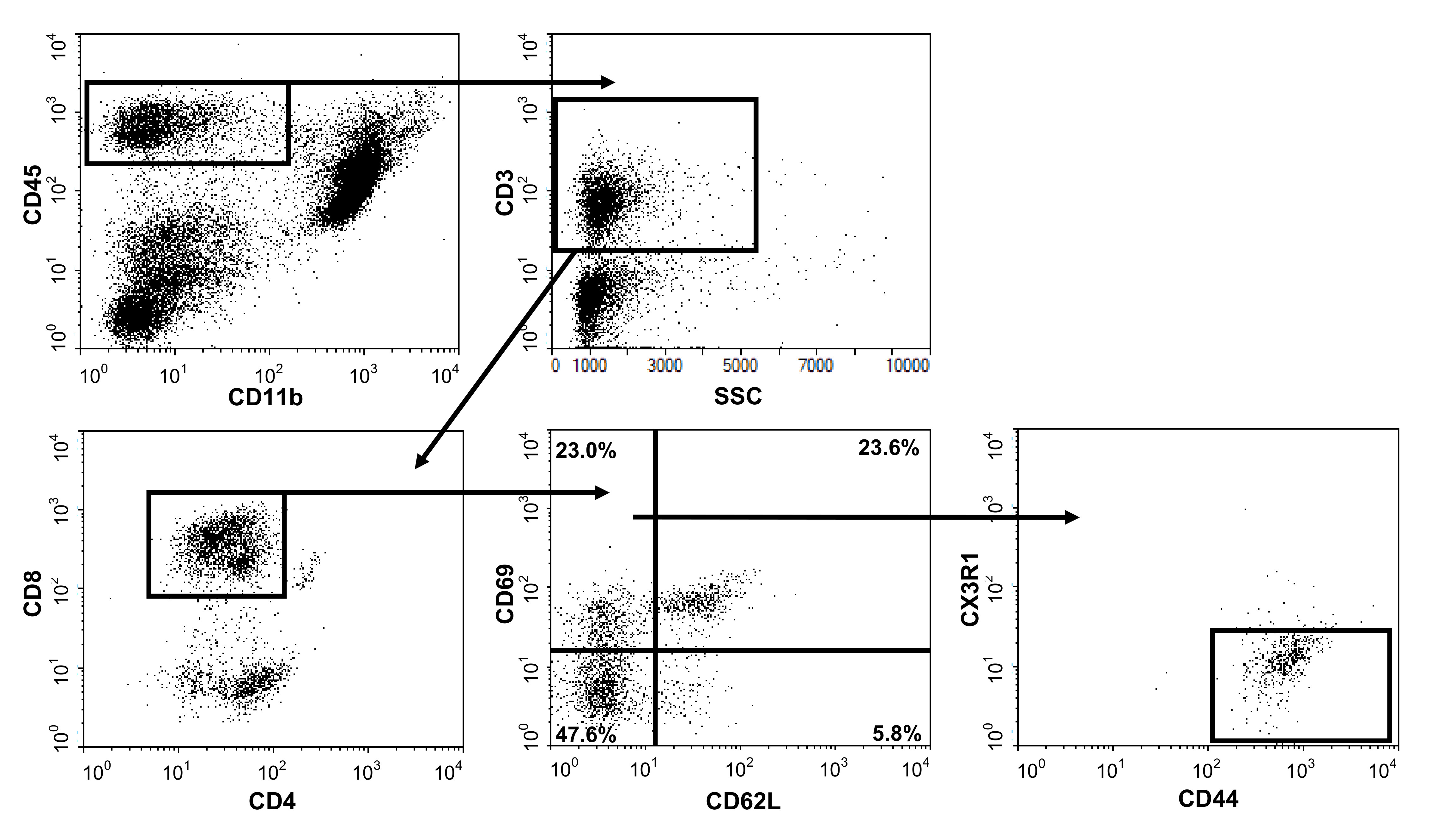

Supplement: Supplementary file 1 — Additional file 1: Supplemental Fig. 1. Gating of brain CD8+ TRM. Representative FACS gating for CD8+ bTRM. Mouse shown was an uninfected 22 mo C57BL/6JN mouse. [file 12979_2022_281_MOESM1_ESM.jpg]

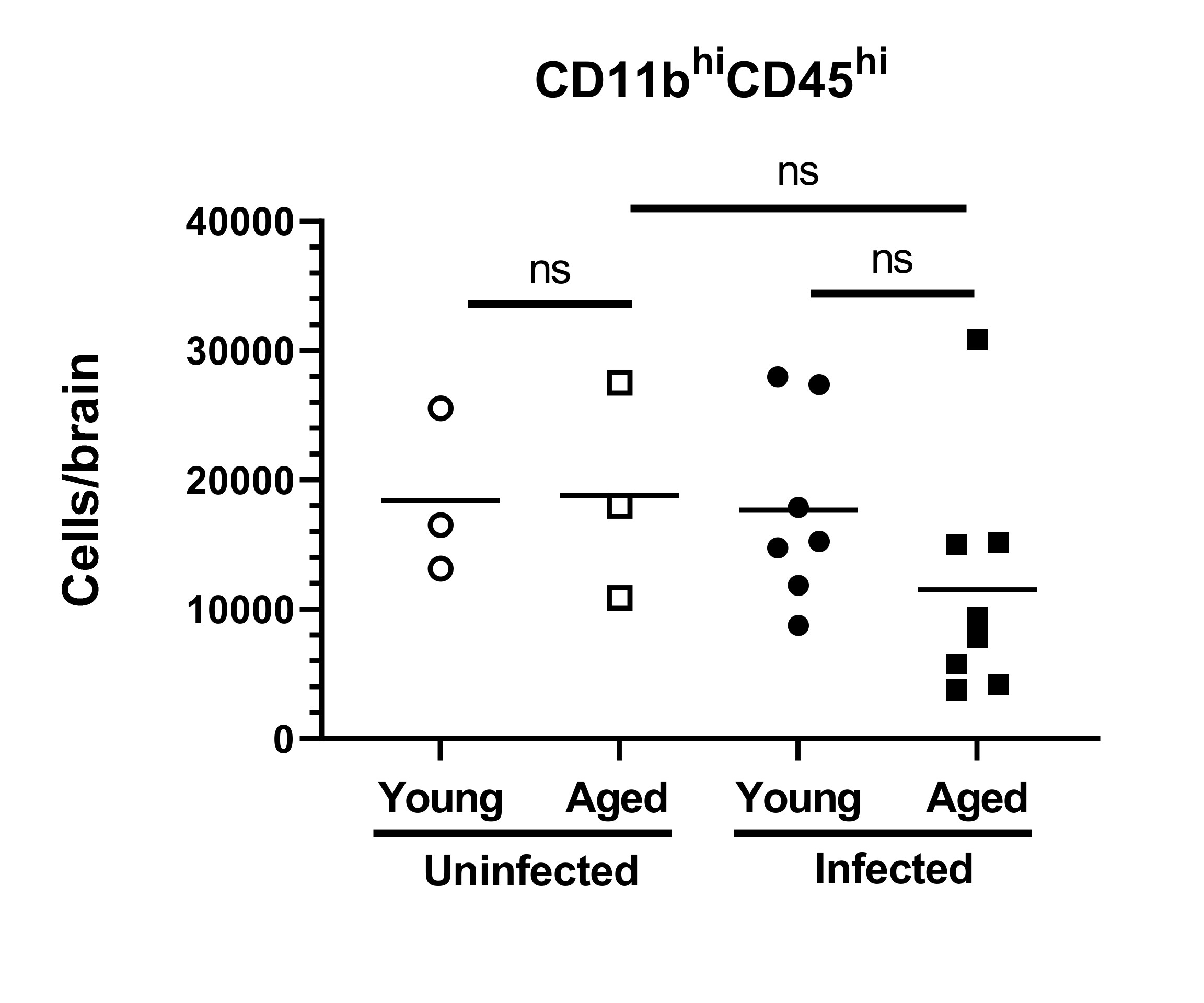

Supplement: Supplementary file 2 — Additional file 2: Supplemental Fig. 2. Young and aged mice have similar numbers of CD11bhiCD45hi brain cells before and after infection. 2 mo C57BL/6 J and 24 mo C57BL/6JN mice were infected i.p. with Lm EGD 2.0 × 10^5^ CFU (n = 9) and 0.3–0.6 × 10^5^ CFU (n = 12), respectively, then received antibiotics beginning 2d p.i. Brain leukocytes were obtained from uninfected mice and from infected mice 29d p.i. by enzymatic digestion then were incubated with mAb and analyzed by flow cytometry. Symbols represent individual young uninfected (○), young infected (●), aged uninfected (□) and aged infected (■) mice. Line indicates mean. Statistical significance calculated via Student’s t test. [file 12979_2022_281_MOESM2_ESM.jpg]

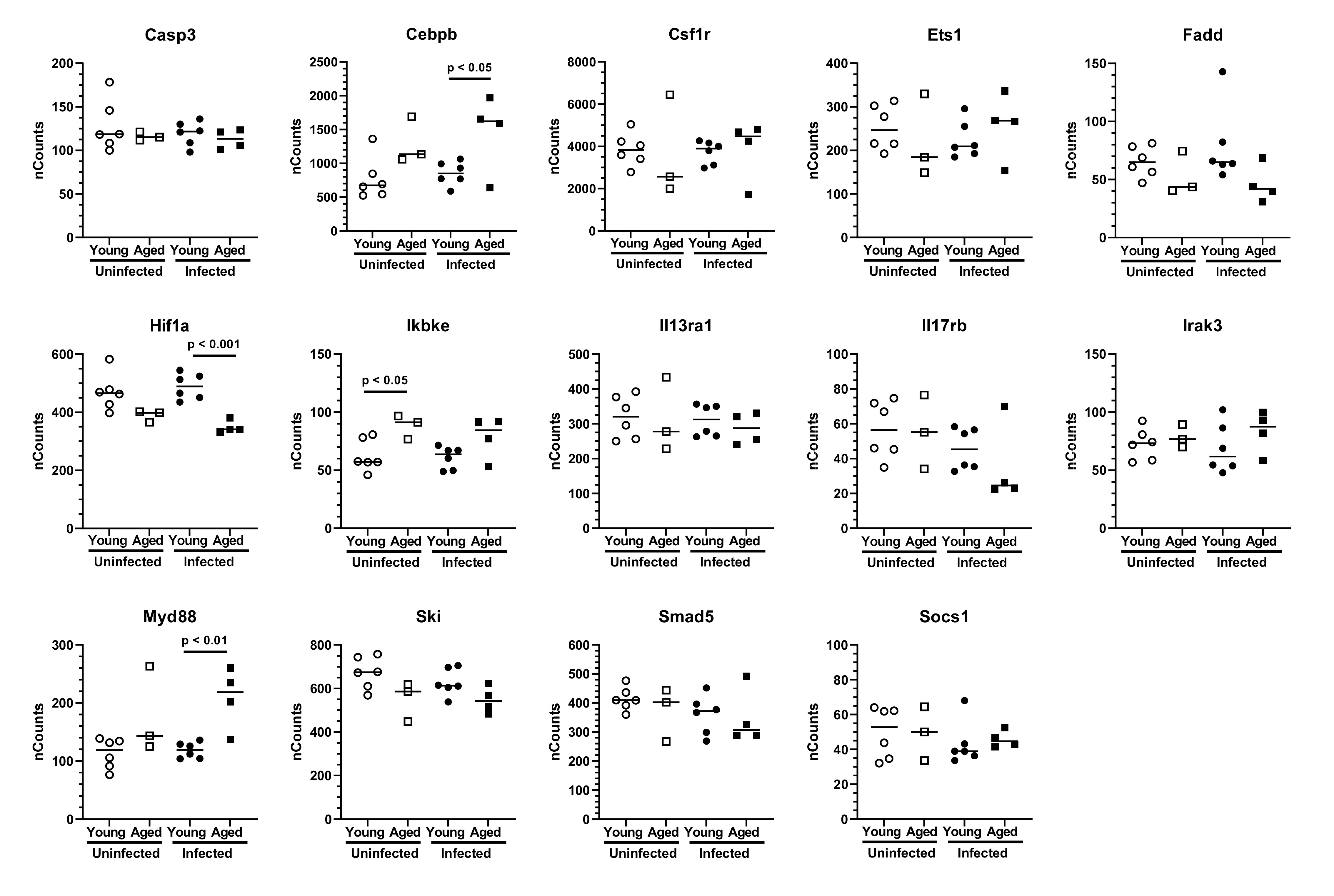

Supplement: Supplementary file 3 — Additional file 3: Supplemental Fig. 3. Transcription factors targeted by miR-155 measured in CD11b+ brain cells from young and aged mice. 2 mo C57BL/6 J and 24 mo C57BL/6JN mice were infected i.p. with Lm EGD 2.0 × 10^5^ CFU (n = 9) and 0.3–0.6 × 10^5^ CFU (n = 12), respectively, then received antibiotics beginning 2d p.i. CD11b+ brain cells were obtained from uninfected mice and infected mice 29d p.i. by magnetic sorting and gene expression was measured by nCounts. Statistical analysis of this restricted list of genes was by simple t-test, a p < 0.05 was considered statistically significant. [file 12979_2022_281_MOESM3_ESM.jpg]

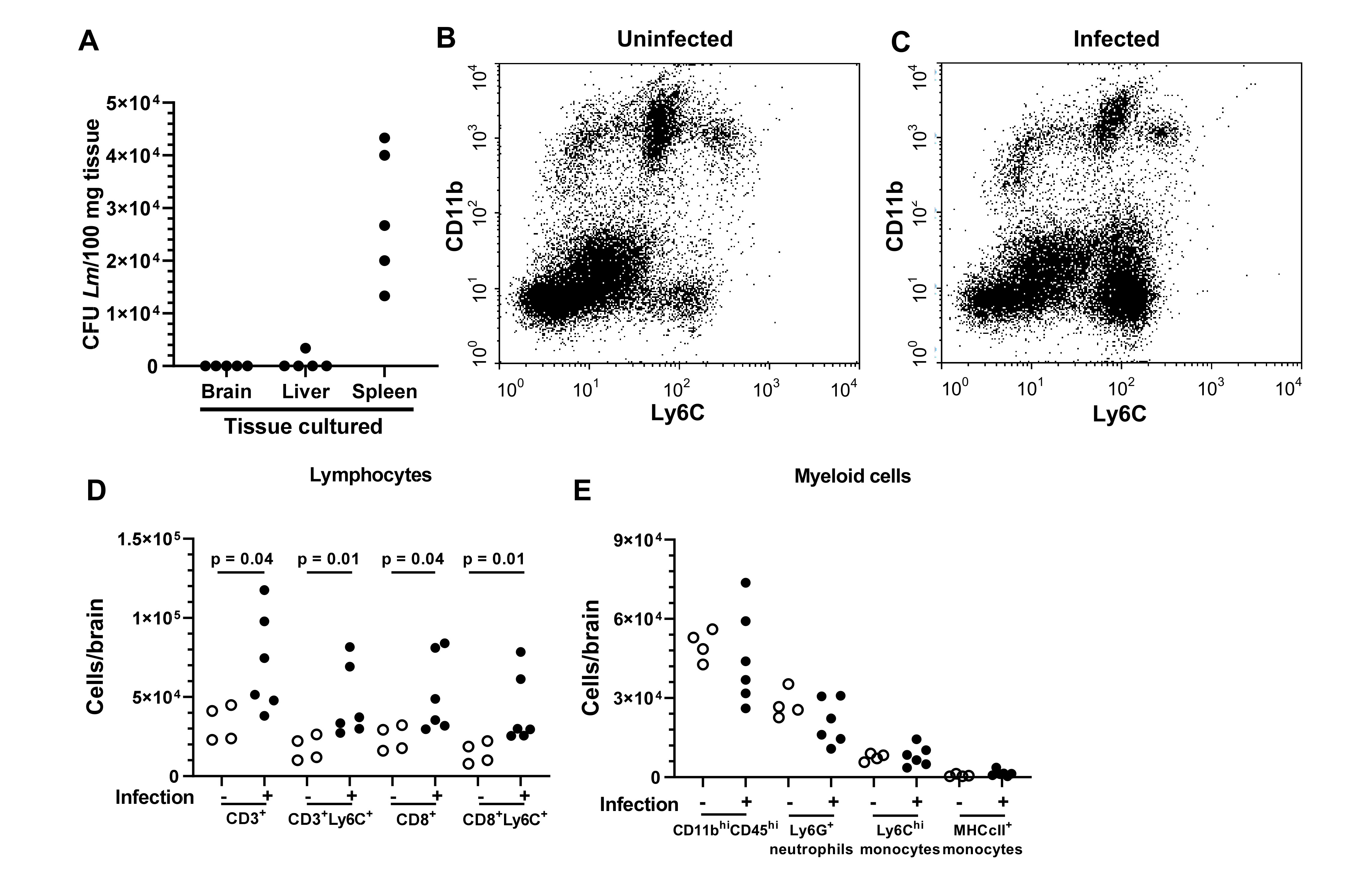

Supplement: Supplementary file 4 — Additional file 4: Supplemental Fig. 4. Preliminary studies of ΔactA Lm infection in aged mice. Panel 20 mo old C57BL/6 J mice (3F, 2 M) were infected with 2.0 × 10^7^ CFU Lm ΔactA (A). Mice were sacrificed D3 p.i., organs were harvested aseptically, homogenized in sterile dH2O, and bacterial CFU were measured by serial dilution and plating on agar. 21 mo C57BL/6JN male mice were infected i.p. with 3 × 10^7^ CFU Lm ΔactA or remained uninfected and given antibiotics starting 2d p.i. (B-E). Mice were harvested 7d p.i. Representative FACS dotplots (20,000 cells/plot) of gated CD45+ cells from uninfected (B) and infected (C) mice at 7d p.i. are shown. Myeloid cells are characterized by CD11b expression, while lymphocytes are essentially CD11b-negative. Data show numbers of brain lymphocytes (D) and myeloid cells (E) cells from individual infected (●) and uninfected (○) mice. Statistical significance between groups calculated by Mann-Whitney U test. [file 12979_2022_281_MOESM4_ESM.jpg]

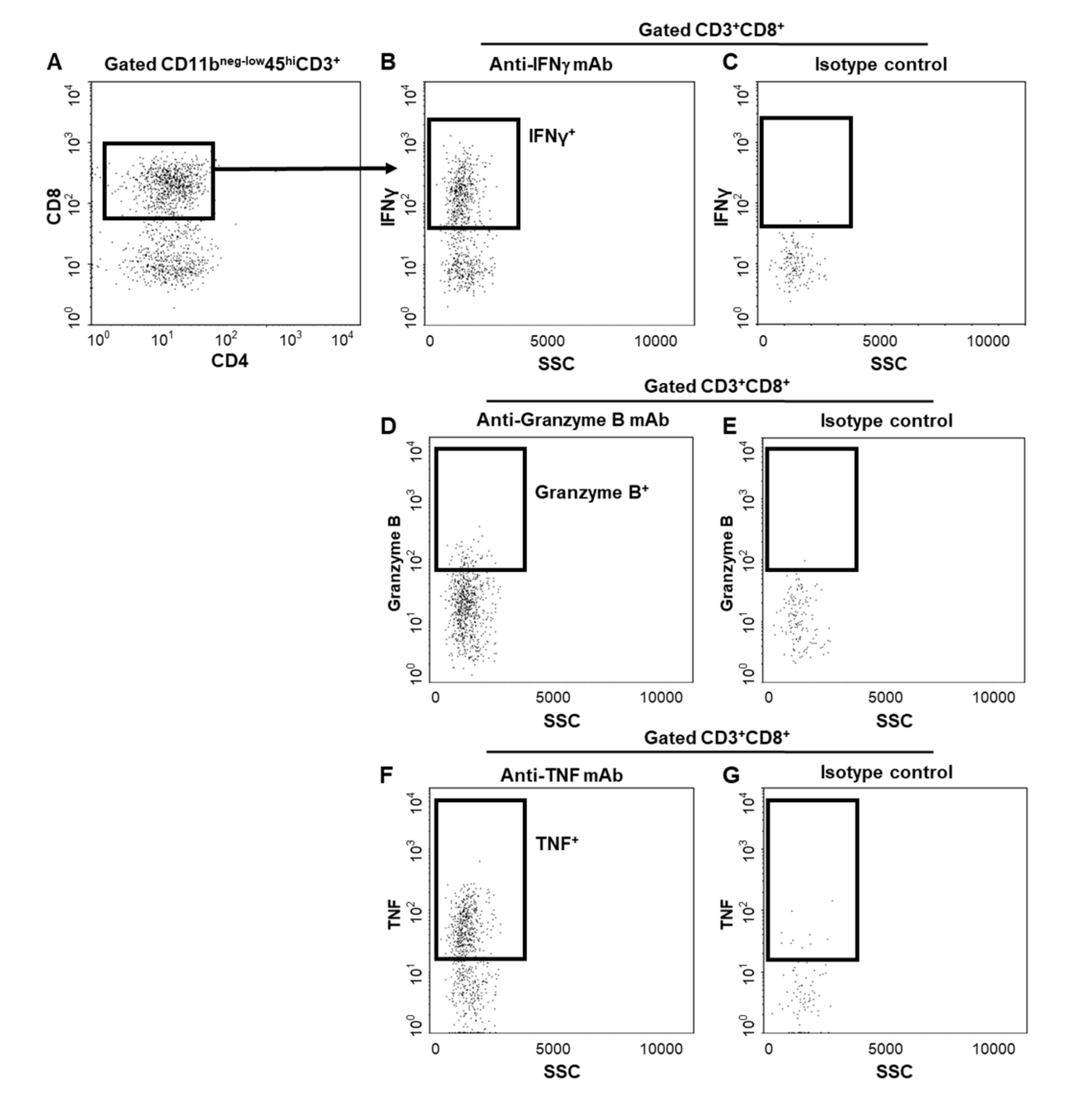

Supplement: Supplementary file 5 — Additional file 5: Supplemental Fig. 5. Gating for intracellular cytokine analysis. Representative FACS dotplots of intracellular cytokine staining as shown in Fig. 6. Dotplots show selection of CD8+ T-lymphocytes from gated brain CD11bneg-low45hiCD3+ cells (A) incubated with anti-IFNg mAb (B) or isotype control (C), which was used to establish expression of IFNγ. Panels (D-G) display similar dotplots for Granzyme B and TNF expression. [file 12979_2022_281_MOESM5_ESM.jpg]
